# VPS13 has an important role in female germline development in Arabidopsis

**DOI:** 10.1111/tpj.70898

**Published:** 2026-05-10

**Authors:** Rosanna Petrella, Camilla Banfi, Vicente Balanzà, Alex Cavalleri, Fabio Radi, Letizia Cornaro, Riccardo Capelli, Carlo Camilloni, Matteo Chiara, Peter J. van Dijk, Diana Rigola, Rik Op den Camp, Mara Cucinotta, Lucia Colombo

**Affiliations:** ^1^ Dipartimento di Bioscienze Università degli Studi di Milano Via Celoria 26 Milan 20133 Italy; ^2^ Instituto de Biología Molecular y Celular de Plantas Consejo Superior de Investigaciones Científicas‐Universitat Politècnica de Valencia Valencia 46022 Spain; ^3^ Keygene N.V. Agro Business Park 90 Wageningen 6708 PW The Netherlands

**Keywords:** female germline, megasporogenesis, ovule development, post‐trascriptional regulation, small RNAs, tasiRNAs

## Abstract

The female germline precursor cell, known as the megaspore mother cell (MMC), differentiates and develops within the ovule through a complex genetic network, including post‐transcriptional and translational regulation mediated by miRNA. A key player in restricting MMC development to a single cell within the nucellus is the *AUXIN RESPONSE FACTOR 3* (*ARF3*). Here, we describe the role of the *VACUOLAR PROTEIN SORTING‐ASSOCIATED 13* (*VPS13*) in post‐transcriptional regulation of *ARF3*. VPS13 is a conserved protein found across all eukaryotes, implicated in membrane dynamics, organelle junctions, and autophagy. We show that in Arabidopsis, the knockout mutation of *VPS13* results in the deregulation of *ARF3, most likely due to a* reduction of tasiR‐ARF production. Consequently, *vps13* mutant ovules exhibit phenotypes similar to those caused by *ARF3* misregulation, such as the formation of multiple MMC‐like cells and defects in megasporogenesis. VPS13 co‐localizes and interacts with the SUPPRESSOR OF GENE SILENCING 3 (SGS3). Based on our findings, we propose an uncharacterized potential role for VPS13 in the homeostasis of membrane‐associated siRNA bodies, essential for tasiR‐ARF biogenesis. Thus, it adds an additional component to the intricate network governing female germline initiation and progression, while also providing new insights into siRNA body homeostasis and tasiR‐ARF pathways. Furthermore, the evolutionary conservation of VPS13 suggests its broader relevance, potentially extending to the understanding of human diseases linked to mutations in its orthologues.

## Significance Statement

This study highlights the role of VPS13 in restricting ARF3 expression within the ovule during megasporogenesis, supporting a model in which VPS13, by interacting with SGS3 and regulating tasiR‐ARF pathway, could be involved in the homeostasis of SGS3 bodies. Thus, the results reported here enhance our understanding of the processes determining female germline progression and suggest broader implications for VPS13's evolutionary conservation.

## INTRODUCTION

In flowering plants, female gametes are produced through a complex developmental process within the ovules, arising from the placenta, a meristematic tissue inside the female reproductive organ. Here, the ovule identity genes, for example, *SEEDSTICK* (*STK*), promote ovule primordia development by negatively regulating the meristematic gene *SHOOTMERISTEMLESS* (*STM*) (Galbiati et al., 2013; Manrique et al., 2024). The female germline precursor cell, named megaspore mother cell (MMC), differentiates in the ovule primordium. We have recently reported that this fundamental process is regulated by a molecular pathway involving SPOROCYTELESS/NOZZLE (SPL/NZZ) and the MADS domain factors STK and SEPELLATA3 (SEP3) to regulate the expression of the auxin transporter PIN FORMED1 (PIN1) (Cavalleri et al., 2026). Following meiosis, the MMC produces a tetrad of haploid megaspores, three of which degenerate, whereas the surviving one will develop into the functional megaspore (FM). Following three rounds of mitosis, the FM will ultimately form a gametophyte that comprises one egg cell and one central cell, which, upon fertilization, will give rise to the embryo and the endosperm, respectively (Hater et al., 2020).

Recent evidence indicates that small RNAs play an important role in restricting female germline fate to a single cell in plants, defining the identity of the female gamete lineage in plants and that this trait might be shared with animals (Cheng et al., 2006; Huang et al., 2022; Olmedo‐Monfil et al., 2010; Pessino et al., 2024; Petrella et al., 2021; Singh et al., 2011). Recently, the miRNA160 has been shown to negatively regulate the expression of its targets *AUXIN RESPONSE FACTOR 10* (*ARF10*) *and 17* (*ARF17*) (Huang et al., 2022; Pessino et al., 2024), whose ectopic expression determines the formation of extra MMC‐like cells. The miR390‐TAS3‐ARF3 module, in particular, is required for restricting female germline fate to a single cell (Su et al., 2017, 2020). Long non‐coding RNAs from the *TAS3* locus are targeted by the miR390‐AGO7 complex to trigger the synthesis of trans‐acting short‐interfering RNAs (tasiRNAs). The cleaved transcripts are then stabilized by SGS3 and converted into dsRNA by RDR6 (Kumakura et al., 2009). The *TAS3*‐derived tasiRNAs (tasiR‐ARF) are successively recruited into an AGO1‐containing effector complex to finally repress the expression of *ARF3* (Fahlgren et al., 2006; Montgomery et al., 2008). Su and collaborators (2017, 2020) showed that pre‐meiotic ovules of mutants lacking *RDR6* or *AGO7* have supernumerary MMC‐like cells and impaired spore degeneration; accordingly, the same phenotype is shown by plants expressing a tasiR‐ARF‐insensitive *ARF3* version in the companion cells surrounding the MMC.

In this study, we characterize the role of *Arabidopsis thaliana VACUOLAR PROTEIN SORTING‐ASSOCIATED 13 S* (*AtVPS13S*)*/SHRUBBY* (*SHBY*; hereafter *VPS13*), which encodes a 3464 amino acid lipid‐binding protein sharing similarity to the yeast Vps13 and human VPS13A, in ovule development (GenBank: AED93352; Levine, 2022). The VPS13 protein has the following domain organization: Chorein‐VAB‐ATG2_C‐PH (Leterme et al., 2023). Homologs of VPS13 have been identified in all eukaryotes and have been proposed to have a broad role in membrane dynamics, organelle junctions, and autophagy (reviewed in Myers & Payne, 2017). In humans, mutations in VPS13‐encoding genes are associated with neurodegenerative diseases such as chorea‐acanthocytosis and Cohen syndrome (Kolehmainen et al., 2003; Lesage et al., 2016; Rampoldi et al., 2001).

In plants, four distinct *vps13* T‐DNA insertion homozygous mutants have been characterized, all showing poor root growth, decreased root meristematic activity, and aberrant cell divisions in root ground tissue (Koizumi & Gallagher, 2013). Furthermore, *vps13* plants are smaller than wild‐type plants, have a shrubby‐like phenotype, and are completely sterile (Koizumi & Gallagher, 2013). Recently, two independent homozygous *vps13* mutant lines (*shby* ^*cr1*^and *shby* ^*cr2*^), with an in‐frame deletion that leads to the formation of truncated proteins, have been reported to have defective root growth and presence of multiple MMC‐like cells in ovules, but adult plant phenotype and final seed set were not described (Cai et al., 2025).

In this study, we characterize the function of VPS13 in megasporogenesis and highlight its critical role in contributing to the regulation of ARF3 through tasiR‐ARF production, which is essential for female germline development. In addition, we discuss the hypothesis of a possible role for VPS13 in tethering SGS3 non‐vesicular bodies required for post‐transcriptional gene silencing (PTGS) mediated by tasiRNAs.

## RESULTS

### *VPS13* is expressed in the ovule and its mutation affects reproductive development

**Figure 1:**
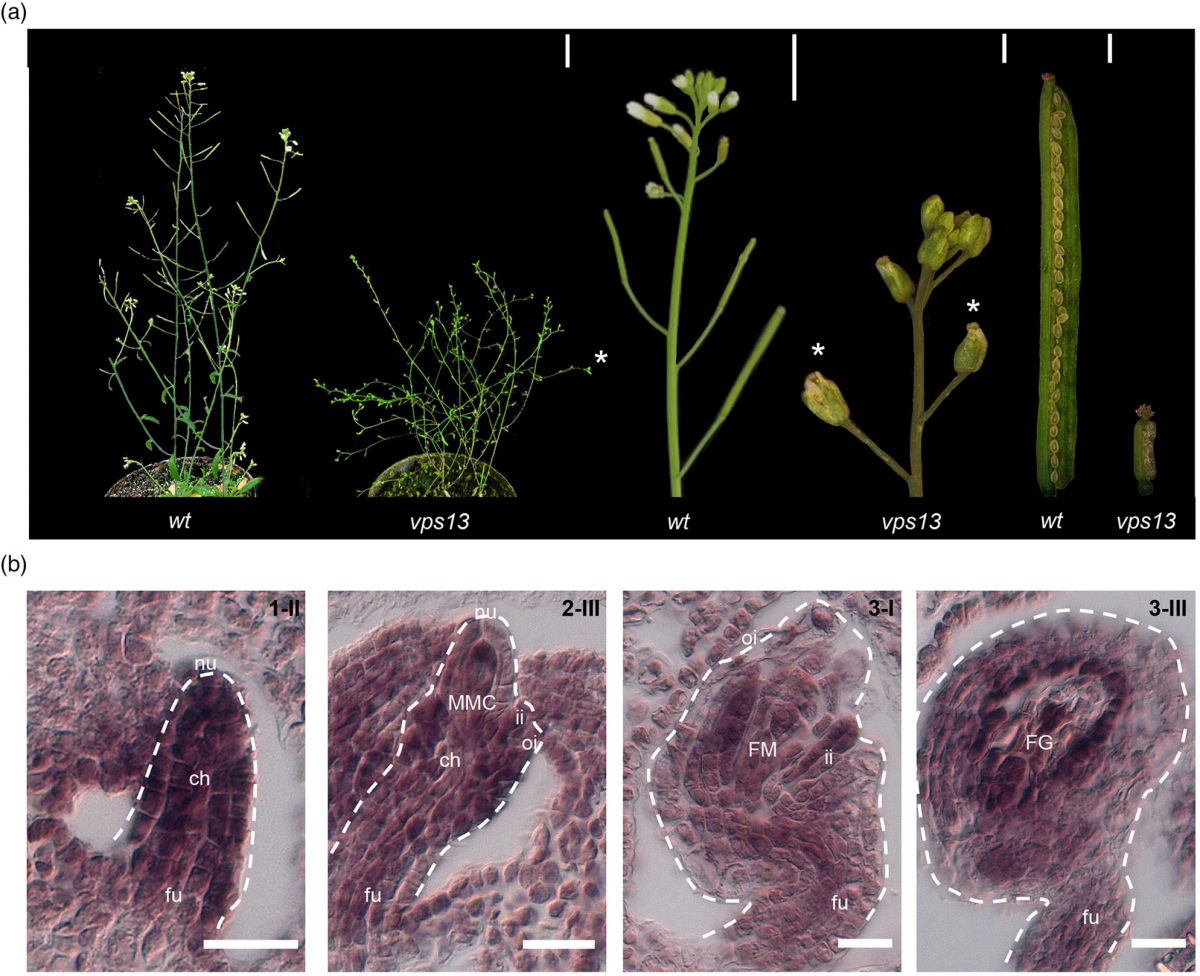
*vps13* mutant phenotype and *VPS13* expression in ovules. (a) From left to right, plant, inflorescence architecture, and seed set of wild‐type and *vps13*. (*) indicates sterile flowers; scale bars = 10 mm. (b) *In situ* hybridization of tissue sections of wild‐type ovules using a *VPS13* antisense probe. The dashed white line indicates the outline of the ovule. ch, chalaza; FG, female gametophyte; FM, functional megaspore; fu, funiculus; ii, inner integument; MMC, megaspore mother cell; nu, nucellus; oi, outer integument; scale bars = 20 μm.

*vps13‐1* (henceforth called *vps13*) mutant plants are smaller than the wild‐type, share a bushy aspect, and present abnormal flowers characterized by pistils containing only aborted ovules, which therefore do not develop into siliques (Figure 1a). An identical phenotype, including complete sterility, has also been observed in two other distinct mutant alleles: *vps13‐3* and *vps13‐4* (Koizumi & Gallagher, 2013). The sterile phenotype was not determined by the defect in the gametophytic haploid phase, since *vps13/+* heterozygous plants were completely fertile. To study *VPS13* expression in ovule development, we performed *in situ* hybridization using a *VPS13*‐specific antisense probe. At stage 1‐II, *VPS13* expression is visible in the primordium; at stage 2‐III, *VPS13* is mainly expressed in the MMC, in the integument primordia, and the funiculus; the *VPS13* transcript subsequently remains detectable in the FM and the inner integuments (3‐I and 3‐III; Figure 1b and Figure S1). These results are consistent with previously reported single‐cell transcriptome data from different domains of the ovule primordium (Hou et al., 2021). In fact, *VPS13* expression was detected in all the ovule domains examined, although its expression level was reported to be the highest in the MMC (Figure S1).

### *vps13* mutation determines the formation of extra‐numerary MMC‐like cells and affects female germline progression

**Figure 2:**
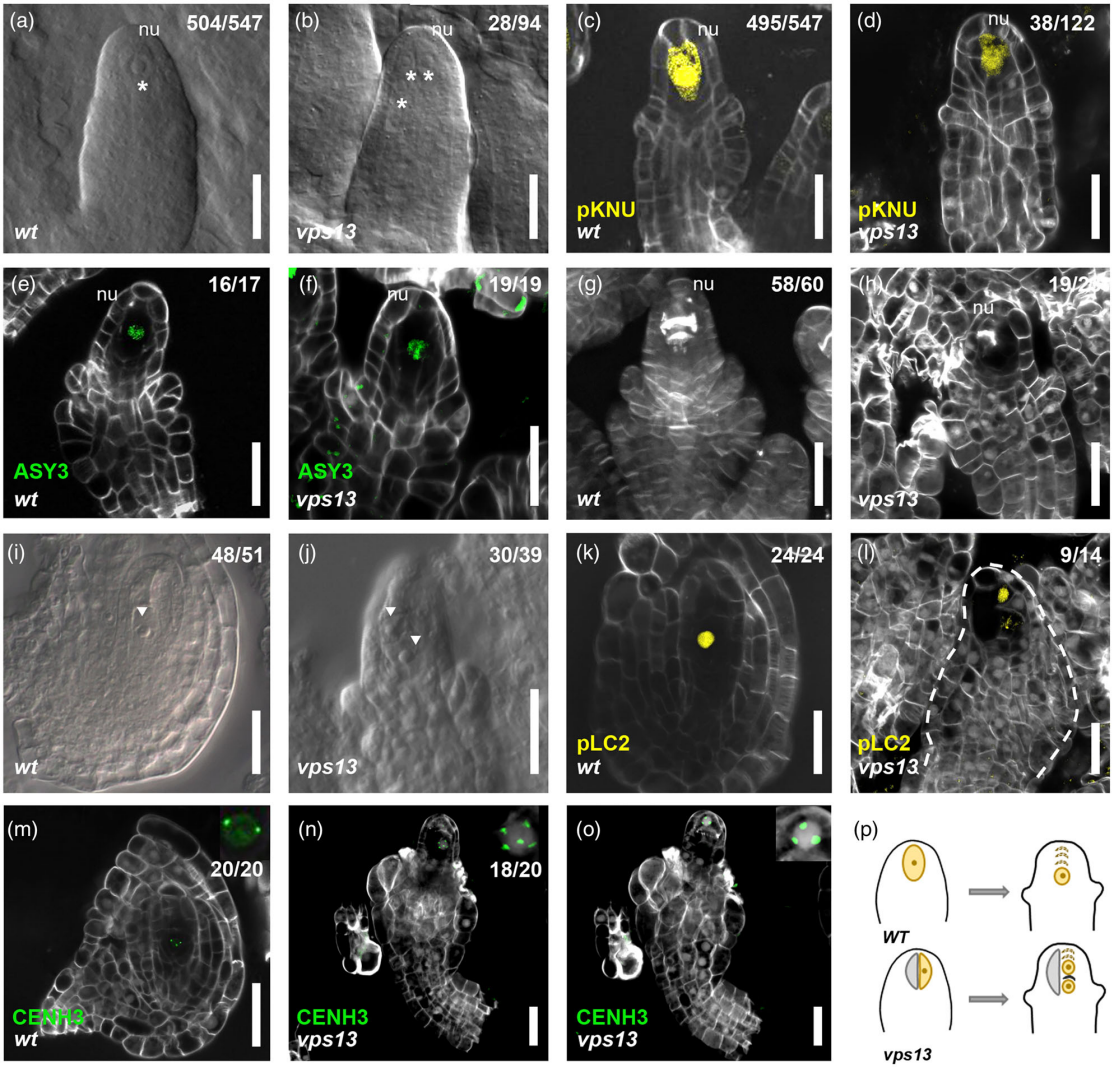
Megasporogenesis defects in the *vps13* mutant. (a, b) DIC images of wild‐type (a) and *vps13* (b) pre‐meiotic ovules; in the wild‐type a single MMC is visible in the nucellus, whereas in *vps13* ovules more than one enlarged cell can be observed, indicated by asterisks. (c, d) *pKNU:nlsYFP* expression in wild‐type and the *vps13* ovules. (e, f). ASY3‐GFP expression in wild‐type (e) and *vps13* (f) pre‐meiotic ovules. (g, h) Callose staining of wild‐type (g) and *vps13* (h) ovules at meiosis; division plates with callose deposition are visible. (i, j) DIC images of wild‐type (i) and *vps13* (j) ovules at stage 3‐I (Schneitz et al., 1995); in the wild‐type, only one spore survives, whereas in the *vps13* ovule, more than one surviving spore is visible (indicated by white triangles). (k, l) *pLC2:nlsYFP* expression in wild‐type and *vps13* ovules, showing more than one cell with a signal in *vps13* compared with the wild‐type. (m–o) *pWOX2:CENH3‐GFP* expression in wild‐type (m) and *vps13* (n–o) ovules at stage 3‐I. (M) Three chromosomes are visible in the figure, while zooming in (the insert) reveals two more chromosomes visible in a different confocal plane. (n–o) Two different confocal planes of the same ovules showing the presence of two cells expressing *pWOX2:CENH3‐GFP*. (p) Cartoon summarizing the megasporogenesis defects of *vps13* mutant. Numbers in figures (a–l) indicate the number of observed phenotypes/total ovules observed. FM, functional megaspore; MMC, megaspore mother cell; nu, nucellus; s, spore; scale bars = 20 μm.

One of the crucial steps of female germline formation is megasporogenesis, which begins when a cell of the nucellus L2 layer enlarges and differentiates to form the MMC. Compared with the wild‐type in which a single MMC is formed (Figure 2a), 30% of *vps13* ovules presented multiple MMC‐like cells, as shown in Figure 2b and Figure S1. To determine whether these cells had MMC identity, we used the *pKNU:nlsYFP* marker (Tucker et al., 2012), specifically expressed in the MMC. The YFP signal was detected in 90% of the MMCs in the wild‐type (Figure 2c), whereas around 30% of the MMCs of the *vps13* mutant ovules expressed *pKNU:nlsYFP* and none of the supernumerary MMC‐like cells yielded a signal (Figure 2d and Figure S1). Despite the *pKNU:nlsYFP* expression, all the MMCs in either of the two genotypes entered meiosis, as demonstrated by the *gASY3‐GFP* marker, which encodes for ASYNAPTIC 3, a coiled‐coil domain protein that is required for normal meiosis (Yang et al., 2019) (Figure 2e,f). Analysis of this reporter line also indicates that none of the supernumerary MMC‐like cells is committed to enter meiosis. Progression through meiosis was evidenced by callose staining that revealed the formation of cell plates between the daughter cells in both wild‐type and *vps13* ovules (Figure 2g,h). It is noteworthy to notice that the dividing MMC was not centrally located in the nucellus in 19 out of 23 *vps13* ovules at the meiosis stage, and this was most likely due to the presence of the additional enlarged cell beside (compare Figure 2g,h). Our result correlates with the percentage of multiple MMC‐like cells recently detected in *shby* ^*cr1*^ and *shby* ^*cr2*^ mutant ovules, and the description that supernumerary MMC‐like cells fail to enter meiosis (Cai et al., 2025).

In angiosperms characterized by monosporic embryo sac development, such as *Arabidopsis*, only the most chalazal spore survives after meiosis to differentiate into the FM, whereas the other three degenerate (Figure 2i). Interestingly, in *vps13* mutant ovules, more than one spore survives after meiosis (Figure 2j). This observation was confirmed using *pLC2:nlsYFP*, which specifically marks the FM (Figure 2k, Tucker et al., 2012). As shown in Figure 2l, more than one spore had the YFP signal in the *vps13* ovule, suggesting defects in megaspore degeneration and FM identity specification.

Despite the above‐described defects, all the *vps13* post‐meiotic spores presented the correct ploidy as shown by introducing the *pWOX2:CENH3‐GFP* reporter line (De Storme et al., 2016) (Figure 2m–o and Figure S1).

To summarize, despite the formation of multiple MMC‐like cells in pre‐meiotic *vps13* ovules, only one differentiates into an MMC and undergoes meiosis. Moreover, although meiosis produces four haploid megaspores, the degenerative process leading to the formation of only one FM is impaired in *vps13*. Then, the gametogenesis process does not initiate in *vps13* ovules (Figure 2p and Figure S1).

### Tissue‐specific activity of *VPS13* in the ovule is important for proper megasporogenesis progression

The *vps13* ovules showed severe defects in integument development (Figure 2j,l,n,o). It has been suggested that the integuments might play pivotal roles in the progression of female germline development (Johnston et al., 2007; Bencivenga et al., 2012; Figueiredo & Köhler, 2014; Petrella et al., 2022). Therefore, to determine whether the phenotype of *vps13* was caused by the role of *VPS13* in the integuments or by its activity in the nucellus, we developed plants that specifically silence *VPS13* expression in the nucellus.

**Figure 3:**
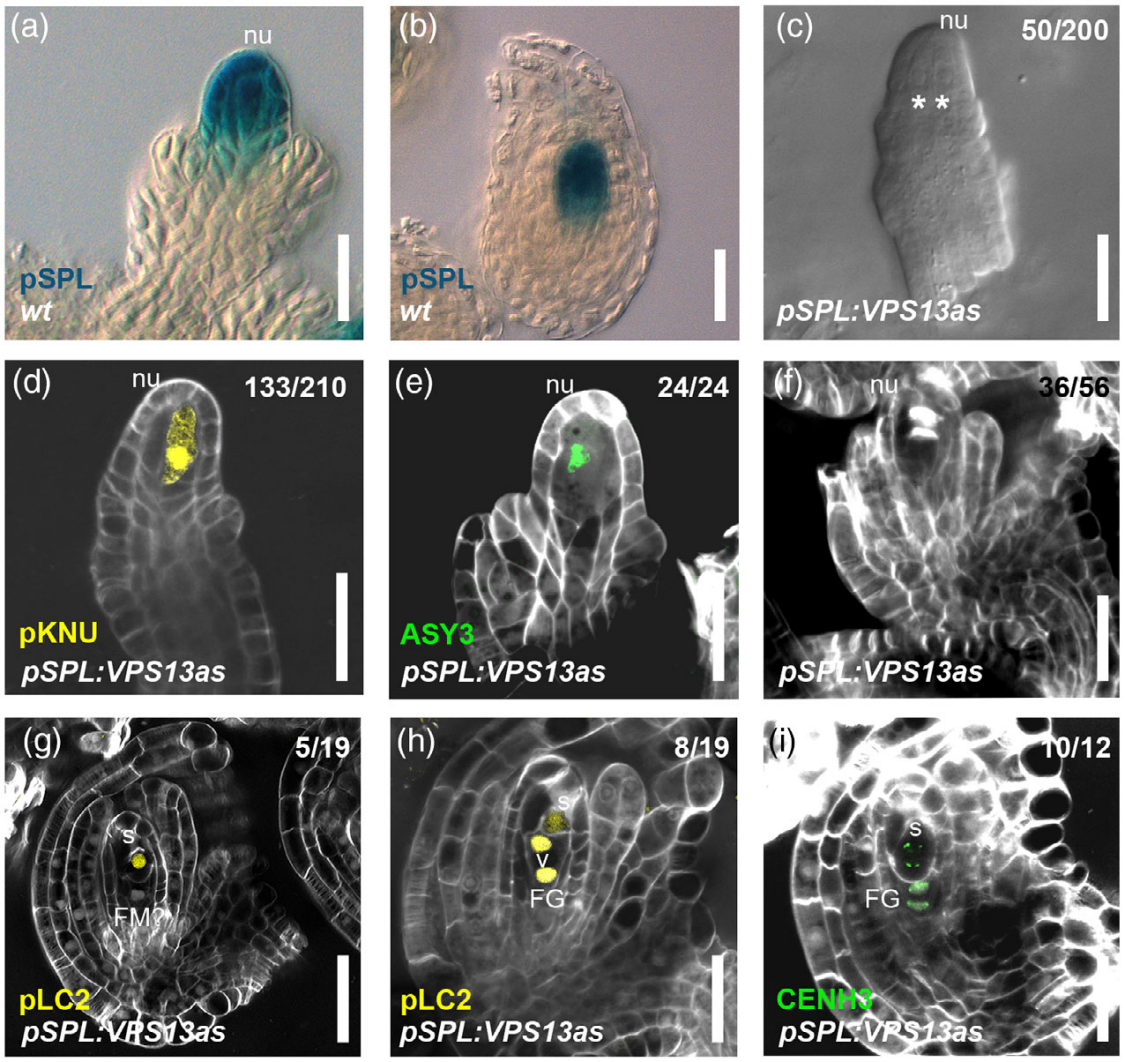
Silencing of *VPS13* in the nucellus phenocopies *vps13* megasporogenesis defects. (a, b) DIC images of ovules expressing *pSPL:GUS:3′UTR*. (c) DIC images of *pSPL:VPS13as* pre‐meiotic ovules, showing two enlarged cells in the nucellus (with asterisks). (d) *pKNU:nlsYFP* expression in *pSPL:VPS13as*. (e) ASY3‐GFP expression in *pSPL:VPS13as* ovules. (f) SR2200 staining of *pSPL:VPS13as* ovules at meiosis. (g, h) *pLC2:nlsYFP* expression in *pSPL:VPS13as*. In (g), there are two surviving spores after megasporogenesis and the most apical one acquires FM identity; in (h) a surviving spore with a faint yellow signal is still visible at the FG2 stage. (i) *pWOX2:CENH3‐GFP* expression in *pSPL:VPS13as*; a spore with a clear signal is still visible at FG2. Numbers in figure C to I indicate the number of observed phenotypes/total ovules observed. FG, female gametophyte; FM, functional megaspore; nu, nucellus; s, spore; v, vacuole; scale bars = 20 μm.

We adopted an antisense approach by using the regulatory region of *SPL/NZZ* (Mendes et al., 2020; Cavalleri et al., 2026; Figure 3a,b) to silence *VPS13* solely in the nucellus. Phenotypic characterization of *pSPL:VPS13as* plants (three independent T1 lines, 8 plants each; Figure S1) showed that while the development of the integuments was not compromised, ovules presented similar defects as observed in *vps13* mutants; indeed, 25% of the observed ovules had more than one enlarged cell in the L2 layer of the nucellus (Figure 3c and Figure S1). The analysis of *pKNU:nlsYFP* (Figure 3d), ASY3‐GFP (Figure 3e), and callose staining (Figure 3f) confirmed the same defects observed in *vps13* knockout line during megasporogenesis. Either the correct establishment of a single FM or the process of spore degeneration was impaired in *pSPL:VPS13as* plants. As a matter of fact, in some ovules, one of the apical spores acquired FM identity (Figure 3g) and showed a persistent signal of the FM marker (Figure 3h). We could confirm these cells to be spores since they showed five chromosomes as expected for meiotic products (Figure 3i). The *pSPL:VPS13as* lines present around 40% ovule abortion, which correlates with the percentage of ovules blocked in the early stages of gametogenesis (Figure S1). In conclusion, the downregulation of *VPS13* expression in the nucellus was sufficient to phenocopy the defects observed in *vps13* knockout mutant ovules during sporogenesis and gametogenesis, even if with lower penetrance, probably as a consequence of residual *VPS13* expression within the nucellus.

### Lack of *VPS13* activity affects ARF3 accumulation in ovules

The defects we have observed in MMC development, leading to impaired megasporogenesis and megagametogenesis in both *vps13* mutants and in *pSPL:VPS13as* plants have been previously described in mutants with defects in the small RNA pathway, especially related to the regulation of *ARF3, ARF10, and ARF17* (Huang et al., 2022; Olmedo‐Monfil et al., 2010; Pessino et al., 2024; Su et al., 2017; Su et al., 2020), and recently to the regulation of the gibberellin and cytokinin signaling pathway (Cai et al., 2025).

**Figure 4:**
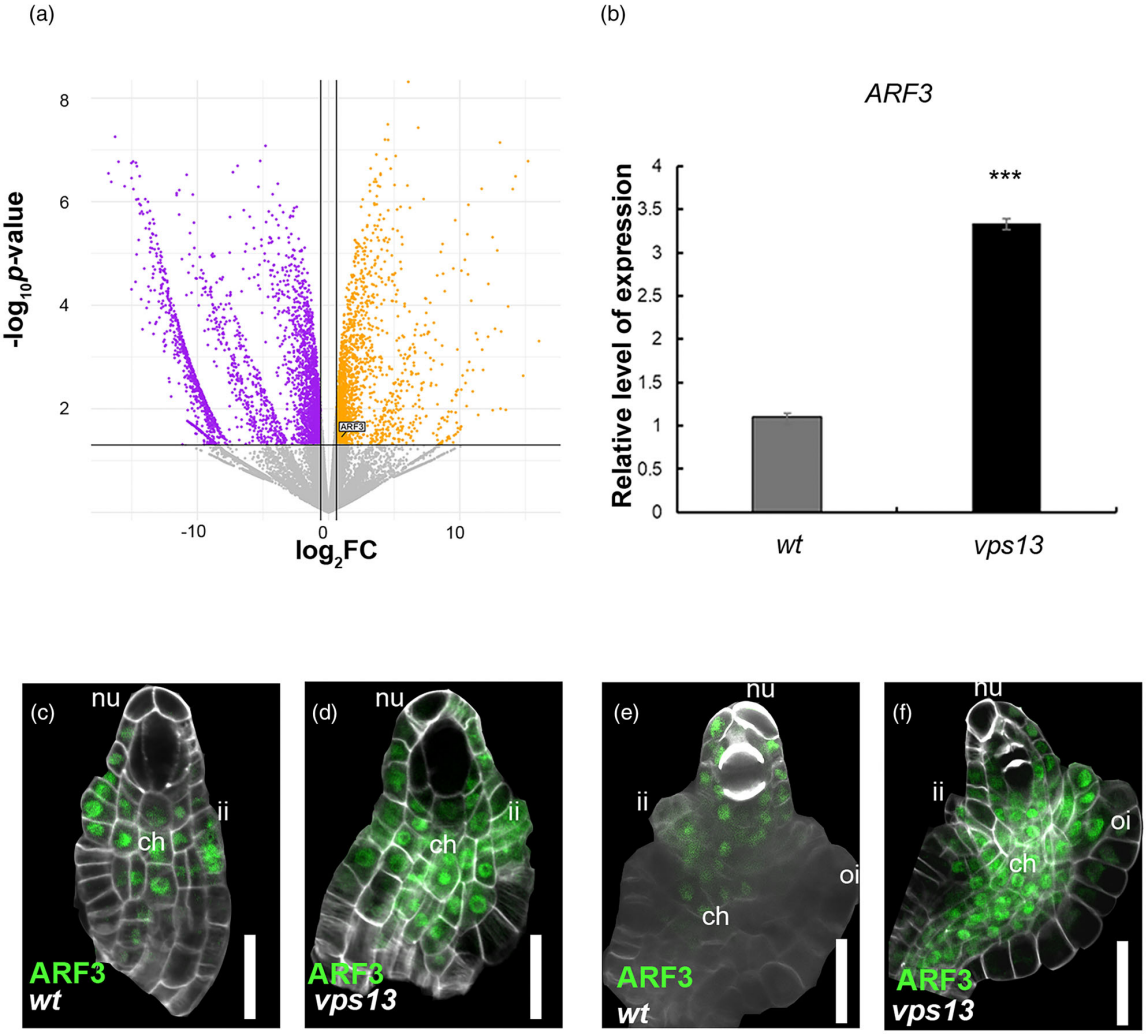
*VPS13* mutation causes *ARF3* upregulation and ARF3‐GFP ectopic accumulation in the ovule. (a) Volcano plot depicting differentially expressed mRNAs in *vps13* compared with wild‐type inflorescences. Orange dots represent mRNAs expressed at higher levels in *vps13* while magenta dots represent mRNAs with lower expression levels in *vps13*. *ARF3* transcript is highlighted among the upregulated genes in *vps13*. The Y‐axis denotes − log10 *P*‐values while the X‐axis shows log2 fold change values. (b) Expression of *ARF3* in wild‐type and *vps13* inflorescences detected by qRT‐PCR. Expression was normalized to that of *UBIQUITIN10* and the expression level in wild‐type was set to 1. ***P < 0.001 (unpaired two‐tailed Student's *t*‐test). (c–f) ARF3‐GFP expression in wild‐type (c, e) and *vps13* (d, f) ovules. In *vps13* ovules, the ARF3 domain is no longer restricted to the chalaza and the lower cells of the L1 layer of the nucellus. nu, nucellus; ch, chalaza; ii, inner integument; oi, outer integument; scale bars = 20 μm.

We therefore decided to investigate whether *VPS13* might play a role in one of these pathways by performing an RNA‐seq analysis on *vps13* and wild‐type inflorescences. Interestingly, among the upregulated genes (log2FC >0.06, *P*‐value <0.05; Table S1), we identified *ARF3* (Figure 4a). As shown in Figure 4b, real‐time PCR analysis confirmed its three times higher expression in *vps13* (3.07 ± 0.77) compared with the wild‐type (1 ± 0.09). To characterize ARF3 protein accumulation in the ovule, we crossed our *vps13* mutant with plants expressing *pARF3:ARF3‐GFP* (Simonini et al., 2016). In the ovule primordium, ARF3‐GFP is present in the chalaza, in the integument primordia, and in the L1 lower cells of the nucellus (Figure 4c). By contrast, we detected a stronger and wider accumulation of ARF3‐GFP in the chalaza of *vps13* pre‐meiotic ovules; more importantly, the domain of expression of ARF3‐GFP expanded across the L1 and L2 layers of the nucellus, as shown in Figure 4d. In wild‐type ovules, ARF3‐GFP level decreased once meiosis started (Figure 4e), whereas it was still detected in the chalaza and the nucellus of *vps13* ovules (Figure 4f).

### *vps13* mutation causes impaired *TAS3* transcript maturation in ovules

**Figure 5:**
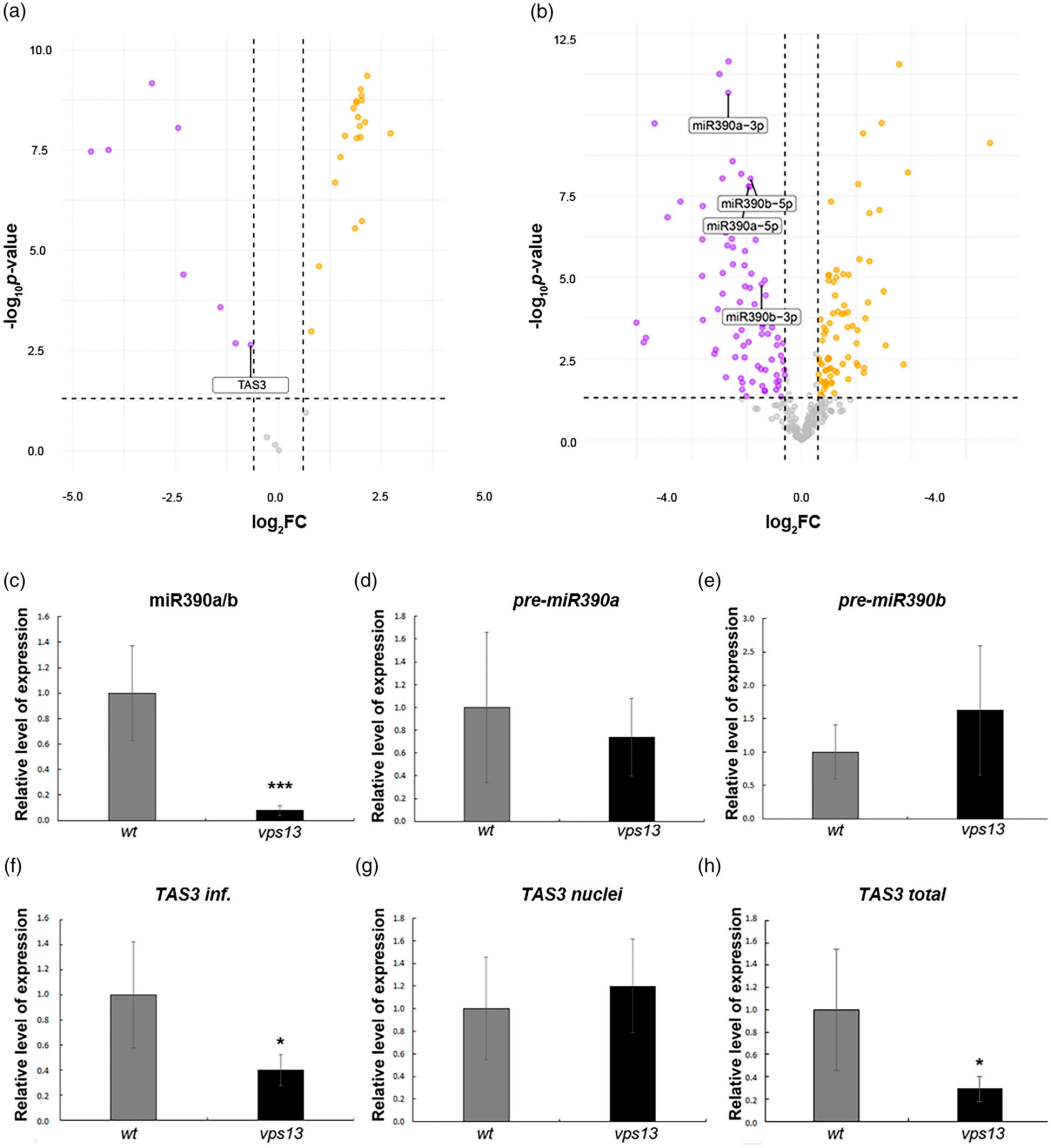
TasiR‐ARF pathway is affected in *vps13* mutant. (a) Volcano plot depicting differentially expressed phasiRNAs and tasiRNAs in *vps13* compared with wild‐type inflorescences. Orange dots represent phasiRNAs and tasiRNAs expressed at higher levels in *vps13*, while magenta dots represent phasiRNAs and tasiRNAs with lower expression levels in *vps13*. *TAS3*‐derived tasiRNAs (indicated as TAS3 in the Figure) are highlighted among the downregulated tasiRNAs in *vps13*. The Y‐axis denotes − log10 *P*‐values while the X‐axis shows log2 fold change values. (b) Volcano plot depicting differentially expressed miRNAs in *vps13* compared with wild‐type inflorescences. Orange dots represent miRNAs expressed at higher levels in *vps13* while magenta dots represent miRNAs with lower expression levels in *vps13*. miR390a/b are highlighted among the downregulated miRNAs in *vps13*. The Y‐axis denotes − log10 *P*‐values while the X‐axis shows log2 fold change values. (c) Stem‐loop PCR expression analysis of *miR390a/b* by qRT‐PCR in wild‐type and *vps13* inflorescences normalized to that of rRNA 5S and the expression level in wild‐type was set to 1. ***P < 0.001 (unpaired two‐tailed Student's *t*‐test). (d–f) Expression of *pre‐primiR90a* (d), *pre‐miR390b* (e), *TAS3* (f), by qRT‐PCR in wild‐type and *vps13* inflorescences. (g, h) Expression of *TAS3* in RNA nuclear fraction (g) and total RNA fraction (h), by qRT‐PCR in wild‐type and *vps13* plants. Expression was normalized to that of *UBIQUITIN10* and the expression level in wild‐type was set to 1. *P < 0.05; ***P < 0.001 (unpaired two‐tailed Student's *t*‐test).

As stated above, the miR390 binding to the *TAS3* transcript drives the production of the 21 nt tasiRNAs (annotated as *PHASI21‐21* by Chen et al., 2021), which in turn silence *ARF3* expression (Fahlgren et al., 2006). Since ARF3‐GFP was ectopically accumulated in *vps13* mutant ovules we performed a small RNA‐seq analysis on *vps13* and wild‐type inflorescences to analyze the level of tasiR‐ARF. Out of the 35 phasiRNAs currently annotated in *Arabidopsis* according to the sRNA database (Chen et al., 2021), 28 were differentially regulated in *vps13* compared with the wild‐type. Specifically, eight of them were downregulated, and among these, TAS3‐derived tasiRNAs showed a significant reduction in expression of 1.6‐fold (FDR <0.05) in the mutant (Figure 5a). Interestingly, short‐read RNA‐seq data showed that TAS3‐derived tasiRNAs are among the most abundant phasiRNAs in the inflorescence, with an average increase of 30X‐fold in the number of assigned reads compared with other phasiRNAs, thus suggesting its importance in reproductive development (Table S1).

Then, the small RNA‐seq dataset was analyzed for miRNAs landscape using the parameters −1 > log2FC > 1, FDR <0.05, and total reads assigned >50. The analysis found 102 miRNAs to be differentially expressed in *vps13* compared with the wild‐type. Specifically, 56 miRNAs were downregulated in the mutant while 46 resulted upregulated (Table S1 and Figure 5b). The miR390a and miR390b were reduced in the *vps13* mutant, as confirmed by stem‐loop qRT‐PCR results (Figure 5c). We also performed qRT‐PCR on *pri‐miR390a* and *pri‐miR390b* to exclude possible deregulation of the precursor transcripts. As shown in Figure 5d,e, we did not detect any statistically significant differences in either *pri‐miR390a* or *pri‐miR390b* transcript levels in *vps13*. Collectively, these results suggested that the reduced levels of miR390a/b observed in *vps13* were not caused by deregulation of their precursors.

In addition to the reduction of miR390, we observed by qRT‐PCR a statistically significant yet slight decrease in *TAS3* expression in *vps13* inflorescences (0.4 ± 0.12) compared with the wild‐type (1 ± 0.42), as shown in Figure 5f, supporting the RNA‐seq data (Table S2). To determine whether the *TAS3* reduction was due to transcriptional or post‐transcriptional regulatory processes, we quantified *TAS3* transcript levels in whole plant RNA samples extracted from isolated nuclei and total RNA, respectively (see Material and Methods). Nuclear *TAS3* RNA has comparable levels in wild‐type and *vps13* (Figure 5g), whereas the total *TAS3* RNA level was statistically lower in the mutant (0.2 ± 0.11) compared with wild‐type (1 ± 0.54) (Figure 5h). This result suggested that the overall *TAS3* transcription in *vps13* (*TAS3* in nuclear fraction) was similar to the wild‐type. Consequently, given the observation that accumulation of total *TAS3* levels was statistically lower in *vps13* mutant, we concluded that *TAS3* abundance and most likely its stability in the non‐nuclear fraction, was affected. This hypothesis is congruent with the lower level of tasiR‐ARF and ectopic accumulation of ARF3‐GFP in *vps13* ovule (Figures 4d,f and 5a).

### VPS13 co‐localizes with the cytoplasmic siRNA bodies components SGS3, RDR6, and AGO7

Previous studies suggested that the endomembranes may play a role in tasiR‐ARF biogenesis in plants since the main players of tasiRNAs biogenesis, such as miR390, AGO7, RDR6, and SGS3 co‐purify with membranes (Jouannet et al., 2012). Specifically, AGO7 congregates with RDR6 and SGS3 in cytoplasmic siRNA bodies that are linked to the endoplasmic reticulum (ER)/Golgi endomembrane system (Jouannet et al., 2012; Kumakura et al., 2009; Schaad et al., 1997; Skog et al., 2008). Given the peculiarity of VPS13 as a protein important for endomembrane tethering (Swan, 2025), we investigated its possible links with the main components of siRNA bodies.

**Figure 6:**
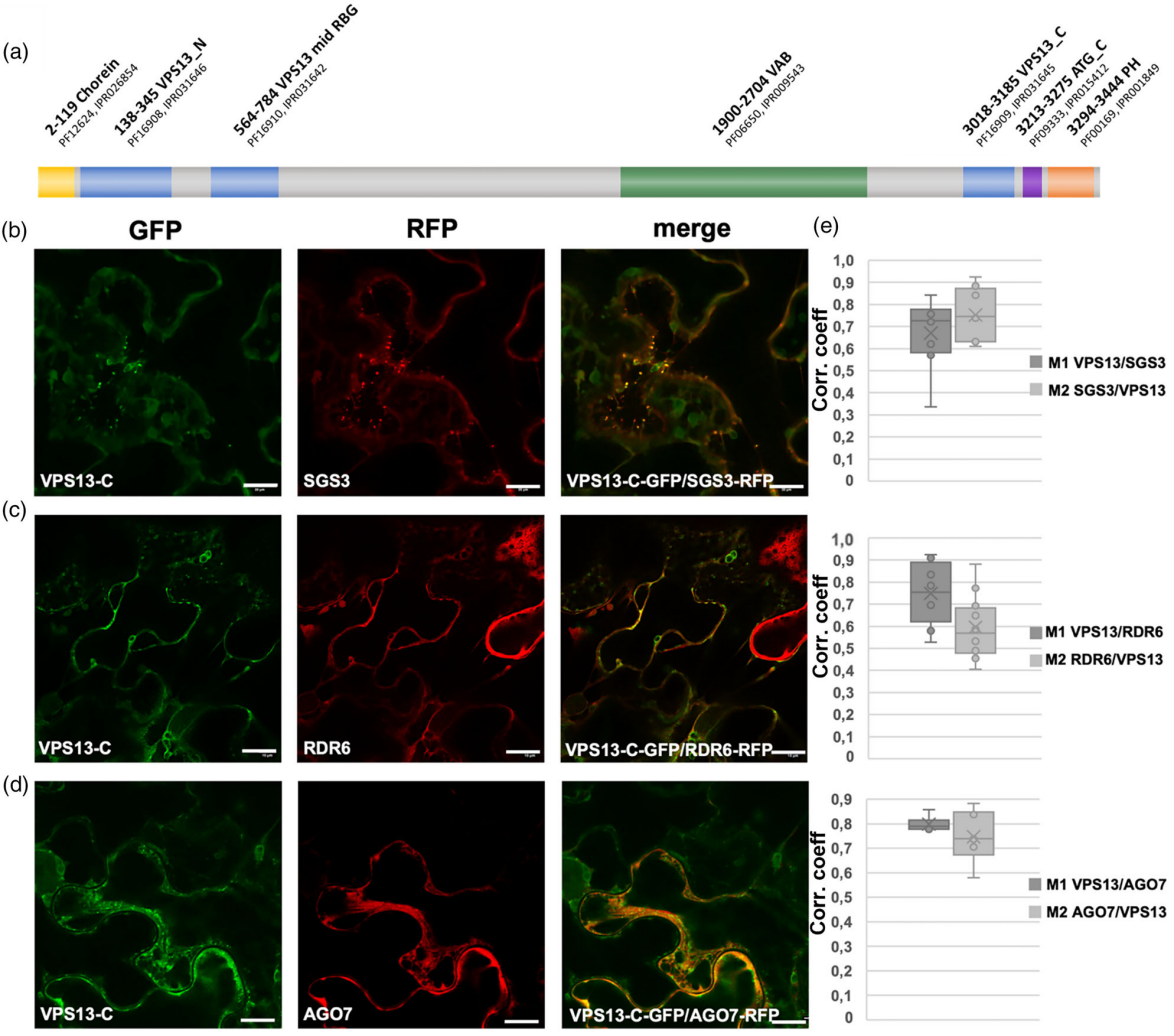
VPS13 co‐localizes with components of siRNA bodies. (a) Schematic model of conserved protein domains in Arabidopsis VPS13. (b–d) Co‐localization experiments in tobacco leaves upon agroinfiltration of VPS13‐C‐GFP and SGS3‐RFP (b), VPS13‐C‐GFP and RDR6‐RFP (c), and VPS13‐C‐GFP and AGO7‐RFP (d). (e) Graphs report the percentage of co‐localization based on the calculation of Manders coefficients; corr. coeff., correlation coefficient. Scale bar, 10 μm.

Thus, we first assessed the percentages of VPS13, SGS3, RDR6, and AGO7 that localized to the ER (Figure S2A–E). For the co‐localization experiment in *N. benthamiana* leaves, we used the VPS13 C‐terminal portion of the protein, containing the membrane contact domains ATG2_C and PH (Figure 6a), and HDEL as a commonly used ER marker (Napier et al., 1992). Co‐expression was assessed by calculating M1 and M2 Manders coefficients as a measure of co‐localization (Dunn et al., 2011) of five different leaf spots infiltrated with VPS13‐RFP/HDEL‐CFP, SGS3‐RFP/HDEL‐CFP, RDR6‐RFP/HDEL‐CFP, and AGO7‐RFP/HDEL‐CFP combinations. In agreement with previous reports, the analysis performed showed a partial overlap between SGS3‐RFP and HDEL‐CFP signals (48%), and between RDR6‐RFP and HDEL‐CFP signals (63%) (Figure S2B,C, respectively). Regarding VPS13, 56% of the VPS13‐C‐RFP signal overlaps with that of HDEL‐CFP, indicating that VPS13‐C is partially associated with the ER, similar to SGS3 and RDR6 (Figure S2A). As expected, a lower level of co‐localization (42%) to the ER was detected for AGO7, suggesting a more dynamic distribution of AGO7 between membranes and the cytoplasm (Figure S2D).

Then, we conducted co‐expression experiments in *N. benthamiana* leaves to verify whether VPS13 physically co‐localizes with SGS3, RDR6, and AGO7 (Figure 6b–d). Either VPS13‐C‐GFP and SGS3‐RFP signals accumulated as small dots that can be interpreted as microbodies associated with the membrane (Figure 6b). Again, we employed M1 and M2 Manders coefficients. The analysis indicates that 67% of VPS13‐C‐GFP signal overlaps with SGS3‐RFP and that 75% of SGS3‐RFP signal overlaps with that of VPS13‐C‐GFP, respectively (Figure 6b). Similar results were obtained for the co‐localization between VPS13 and RDR6 with 75% of VPS13‐C‐GFP signal co‐localizing with that of RDR6‐RFP (Figure 6c). High values of co‐localization also resulted in the VPS13 and AGO7 combined expression (Figure 6d). The correlation coefficients for each tested co‐localization are reported in Figure 6e.

### VPS13 can interact with SGS3

**Figure 7:**
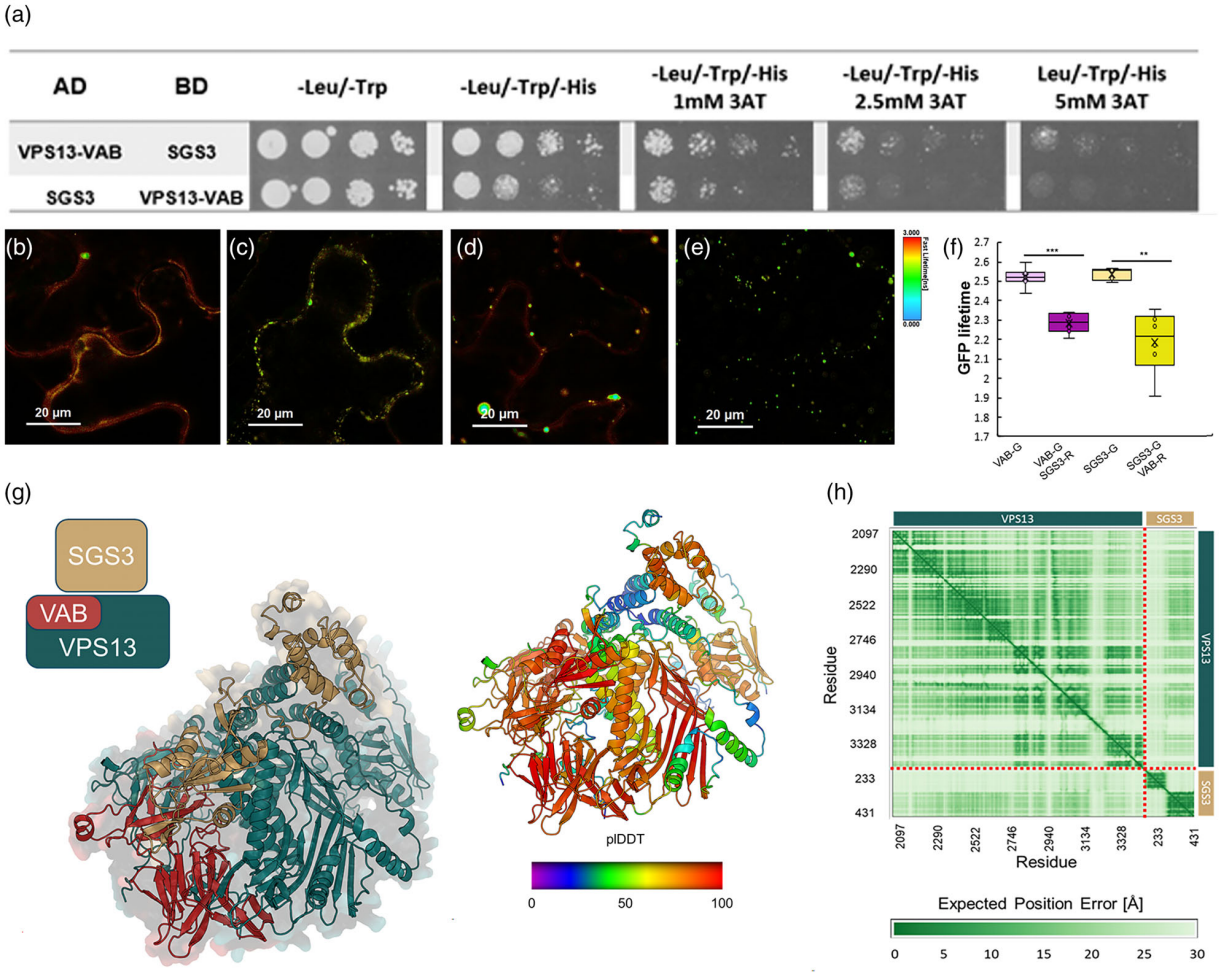
VPS13 can interact with SGS3 *in vivo*. (a) Yeast two‐hybrid assay to test VPS13‐VAB and SGS3 interaction in different selective media. The strength of interaction was tested with different concentrations of 3AT on ‐L ‐W ‐H selective media and four serial dilutions of yeast cells. (b–e) LUT images of VPS13‐VAB‐GFP alone (b), VPS13‐VAB‐GFP with SGS3‐RFP (c), SGS3‐GFP (d), and SGS3‐GFP with VPS13‐VAB‐RFP (e). (f) Graph showing GFP lifetime measured in cells expressing VPS13‐VAB‐GFP or SGS3‐GFP alone compared with those measured in cells co‐expressing VPS13‐VAB‐GFP or SGS3‐GFP together with SGS3‐RFP or VPS13‐VAB‐RFP, respectively. (**) *P*‐value, 0.01 and (***) *P*‐value, 0.001 (unpaired homoscedastic two‐tailed Student's *t*‐test). (g) Best AlphaFold predictions for VPS13–SGS3 complex. Proteins are shown using the cartoon representation. On the left side are the colors of the protein arrangement in the complex. On the right side, the colors show the pLDDT score of the prediction (from red—most confident to blue—less confident): it is worth noting that the less reliable part of the prediction is the one relative to the disordered parts of SGS3 and VPS13. (h) 2D‐Plot shows the predicted aligned error (PAE) for AlphaFold3 predictions for the VPS13–SGS3 complex.

To confirm direct interaction between VPS13 and the protein components of siRNA cytoplasmatic bodies, we performed a yeast two‐hybrid assay. The VPS13 adaptor‐binding domain (VPS13‐VAB; Figure 6a), specialized for protein–protein interaction, can physically interact with SGS3 in yeast (Figure 7a, Figure S3A). On the contrary, no interaction was detected for VPS13 and either AGO7 or RDR6 in yeast (Figure S3B,C).

The interaction between SGS3 and VPS13 was confirmed by performing a FRET‐FLIM analysis on *Nicotiana benthamiana* leaves, testing potential interaction between VPS13 VAB domain (VPS13‐VAB) and SGS3 (Figure 7b–f; Figure S4E–L). The VPS13 C‐terminal (VPS13‐C) domain was used as a control, since it co‐localizes with SGS3 (Figure 6b,e) but it lacks the interactor domain (Figure S4A–D,M–U). We observed a statistically significant decrease in GFP lifetime when either SGS3‐GFP/VPS13‐VAB‐RFP and SGS3‐RFP/VPS13‐VAB‐GFP were tested (Figure 7f); conversely, no decrease was registered in GFP lifetime of SGS3‐GFP when it was expressed with VPS13‐C‐RFP and vice versa (Figure S4U). Thus, these results suggest that VPS13 can physically interact with SGS3 *in planta*, via its VAB domain.

Finally, to gain molecular‐level insight into possible complexes formed by VPS13 together with SGS3, we performed a machine learning‐based structure prediction using the AlphaFold3 web server (Figure 7g,h). We first queried the sequences of the complex components from the UniProt database (https://www.uniprot.org/), resulting in a prediction for 5075 residues. Such a large number of amino acids, combined with the fact that large portions of the sequences are intrinsically disordered regions, reduced the probability of an accurate structure prediction. For this reason, we considered a smaller portion of VPS13, residues 2097–3464, which includes the VAB and VPS13_C domains. In this way, we focus only on the unannotated part of VPS13 protein that is most likely to interact with SGS3. Furthermore, in preliminary runs of the algorithm, we noticed the presence of two large groups of unstructured residues at both the N‐ and C‐terminal side ends of SGS3 (rr. 1–175 and rr. 432–625). The presence of such a disordered region (also predicted by UniProt) added noise to the algorithm and did not allow to obtain a reliable structure. Similarly, we identified a region predicted to be disordered in VPS13 (rr. 2361–2429). We then consistently truncated these regions and ran the structure prediction algorithm, obtaining the structures shown in Figure 7g. The VPS13–SGS3 complex has an average pLDDT score of 63/100 for the best candidate, with most of the less reliable residues in the disordered regions of the modeled portions of SGS3 and VPS13, far from the protein–protein interface. From the positional error matrix (Figure 7h), VPS13 is globally represented with reasonable confidence. Considering SGS3, the second domain (rr. 299–437) is predicted with low positional error (and the same secondary and tertiary structures in all models), directly interacting with the VAB domain of VPS13. The first domain of SGS3 is predicted with less confidence (and less agreement between different models), but it remains in contact with VPS13.

### VPS13 has a potential role in maintaining SGS3 membrane‐less bodies homeostasis

**Figure 8:**
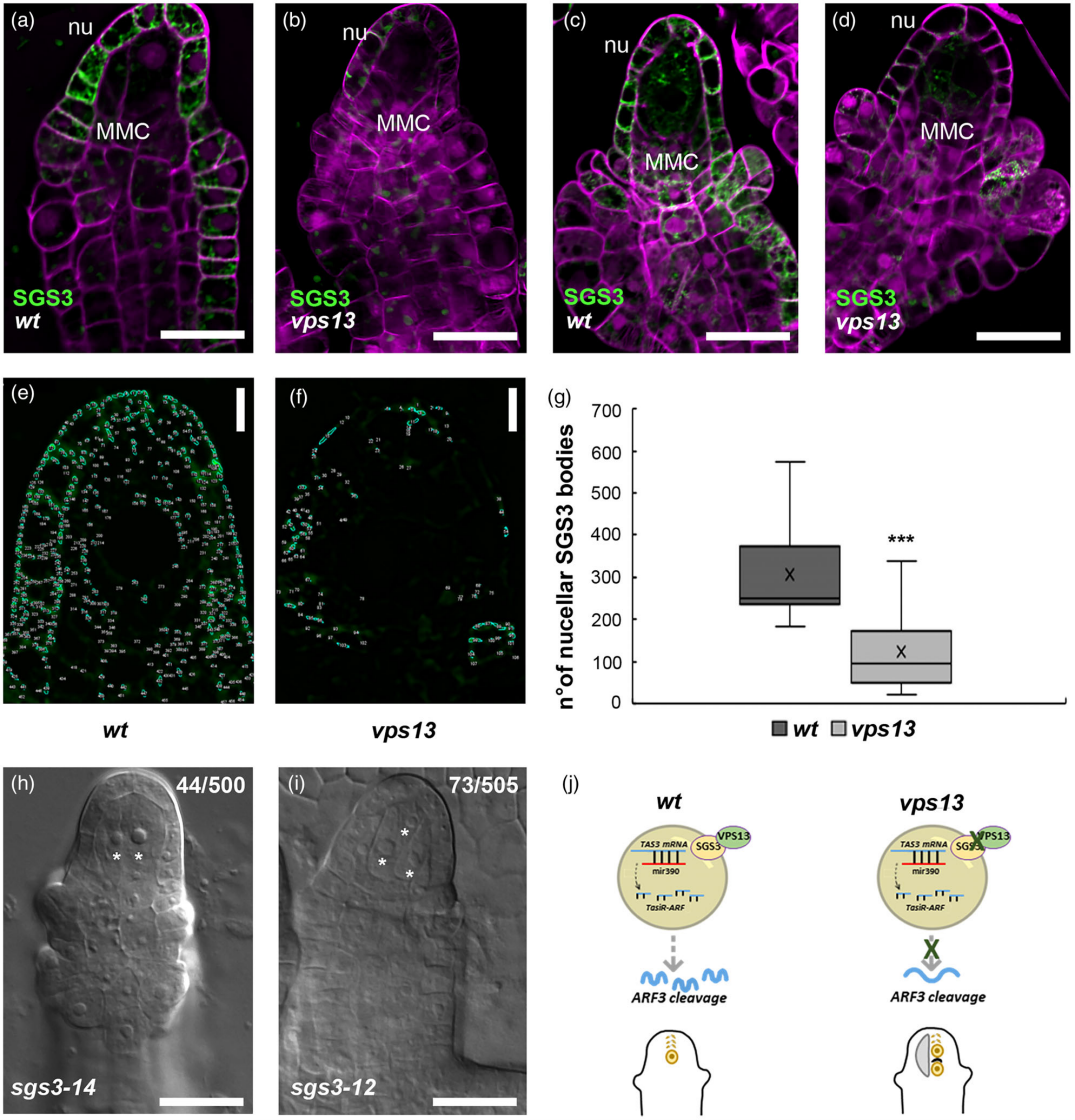
VPS13 promotes the formation of SGS3 bodies. (a, d) *pSGS3:SGS3‐GFP* expression in wild‐type (a and c) and *vps13* (b and d) ovules at two stages of MMC differentiation and development (c and d; e and f). *vps13* presented a drastic decrease in SGS3 expression. (e, f) Nucellus of wild‐type and *vps13* ovules, respectively, expressing *pSGS3:SGS3‐GFP*. SGS3‐GFP has been used as a marker to quantify siRNA bodies, indicated by individual numbers, using Fiji software. (g) The box plot shows the number of nucellar SGS3‐GFP bodies in *n* = 17 (wild‐type) and *n* = 14 (*vps13*) ovules. ***P < 0.001 (unpaired two‐tailed Student's *t*‐test). (h, i) DIC images of *sgs3‐14* (g) and *sgs3‐12* (h) ovules. Multiple MMC‐like cells were indicated with white asterisks. (j) Cartoon summarizing the proposed activity of VPS13 in modulating tasiR‐ARF regulation. Deregulation of *VPS13* expression results in a failed cleavage of *ARF3* transcripts and reported defects in female germline development. MMC, megaspore mother cell; nu, nucellus. Scale bars in (a–d), (h, i) 20 μm; in (e, f) 10 μm.

To verify the potential role of VPS13 on SGS3 bodies formation, we introduced the *pSGS3:SGS3‐GFP* (Su et al., 2020) reporter line in our *vps13* mutant and then compared the SGS3‐GFP accumulation in *vps13* and wild‐type ovules (Figure 8a–d). At MMC differentiation stage, SGS3‐GFP is mainly expressed in the ovule L1 layer, where it congregates in small bodies (i.e., SGS3 bodies) (Figure 8a). Later on, before the MMC enters meiosis, we observed SGS3 bodies also inside the germline cell, but not in the companion cells (Figure 8c). SGS3‐GFP accumulation is drastically reduced in *vps13* ovules, suggesting that the VPS13 function might be important for SGS3 bodies homeostasis at the ER (Figure 8b,d). Using Fiji software, we have quantified the number of SGS3‐bodies in the nucellus of wild‐type (*n* = 17) and *vps13* (*n* = 14) ovules at MMC stage (Figure 8e,f, respectively). As shown in Figure G, *vps13* nucella presented a drastic and statistically significant decrease of SGS3‐cytoplasmatic bodies, compared with the wild‐type. Collectively, these results suggest a supporting role for VPS13 in SGS3 bodies formation and/or stability.

Finally, the analysis of two *sgs3* mutant lines (*sgs3‐14* and *sgs3‐12*) (Peragine et al., 2004) showed that 9 and 13% of ovules, respectively, bear multiple MMC‐like cells, suggesting that lack of *SGS3* activity leads to phenotypic defects during female germline establishment and progression, similar to the observed *vps13* ovule phenotype (Figure 8h,i; Figure S4V).

In conclusion, we proposed a model in which VPS13 by interacting with SGS3, modulates SGS3 bodies abundance in the ovule, that in turn are required for the correct tasiR‐ARF processing and consequently for *ARF3* silencing (Figure 8j).

## DISCUSSION

Small RNAs and tasiRNAs play an important role in plant female germline development (Huang et al., 2022; Olmedo‐Monfil et al., 2010; Pessino et al., 2024; Su et al., 2017, 2020). Mutants for genes required for tasiRNA biogenesis, such as *rdr6* and *ago7*, show a similar phenotype to that of *vps13* with extra‐numerary MMC‐like cells in pre‐meiotic ovules and persisting spores at the end of megasporogenesis (Su et al., 2017, 2020). Therefore, we have here investigated a link between VPS13 and the tasiR‐ARF pathway. Our results revealed that the lack of VPS13 activity causes a reduction in SGS3 bodies, likely impacting the production of tasiR‐ARF that specifically regulate *ARF3*. In fact, we observed that ARF3‐GFP is ectopically accumulated in *vps13* ovules with respect to the wild‐type, suggesting impairment in its post‐transcriptional regulation. The ARF3‐GFP, indeed, was no longer confined to the chalazal region within the ovule, but it expanded to the surrounding domains in the MMC companion cells and the integuments, hence determining the megasporogenesis defects, as already reported by Su et al. (2017, 2020) and observed also in *vps13* ovules in this study. Supporting our findings, we have observed similar defects concerning the restriction of a single female germline precursor in *vps13* and *sgs3* mutants.

ARF3 belongs to the auxin response factor family that regulates the expression of auxin‐responsive genes, and it is known to function in auxin‐regulated pathways, such as pistil development (Simonini et al., 2016). It has also been reported that ARF3 confers floral meristem determinacy by regulating the expression of *WUSCHEL* (*WUS*) (Liu et al., 2014). To broaden the investigation of how *ARF3* expression in ovules led to the multiple MMC phenotype and inhibited spore degeneration, Su et al. (2020) performed RNA sequencing to identify potential downstream genes of ARF3 in ovules at MMC stage. Interestingly, genes associated with metabolism and plant hormone signal transduction pathways (e.g., auxin and cytokinins) were identified as putative targets, corroborating the hypothesis of an involvement of phytohormones in megasporogenesis. The connection between small RNAs and auxin signaling in this process was further reinforced by the recent discovery of the role of miR160 in regulating *ARF17*, that when ectopically expressed regulates the expression of the auxin transporter encoding gene *PIN1* in the ovule nucellus to assure MMC identity (Huang et al., 2022).

The association of *VPS13* with GA and cytokinin pathways (Koizumi & Gallagher, 2013; Cai et al., 2025) suggests the involvement of *VPS13* in other phytohormone responses, which would be intriguing to investigate in more detail.

Although we have observed a lower level of *TAS3*‐derived tasiRNAs in the *vps13* inflorescences, the levels of *TAS1* and *TAS2*‐derived tasiRNAs resulted to be higher. Whether this is a secondary effect of *vps13* mutation or a specificity of VPS13 function for tasiR‐ARF remains an open question. The mir390 is uniquely adapted to initiate *TAS3*‐derived tasiRNA biogenesis due to its specific association with AGO7; on the other hand, *TAS1* and *TAS2* processing are completely dependent on AGO1 (Adenot et al., 2006; Montgomery et al., 2008). Here, we observed a high co‐localization of VPS13 with AGO7, RDR6, and SGS3, main component of SGS3 bodies. In addition, the VPS13‐C‐terminal portion showed similar behavior to that of RDR6 and SGS3 in terms of association with an ER marker.

Accordingly, our analysis showed that the lack of VPS13 activity affects the number of SGS3 bodies, marked by SGS3‐GFP protein expression, in ovules during megasporogenesis. In light of our results, we propose that VPS13 may mediate the formation of membrane‐associated SGS3 bodies or promote their stability to drive the correct biogenesis of *TAS3*‐derived tasiRNAs. In turn, the reduced homeostasis of the SGS3 bodies in *vps13* mutant might affect the stability of the *TAS3* transcript, leading to its reduction. Interestingly, it has been recently reported that SGS3 might have a role in protecting RNAs from degradation (Elmayan et al., 2025). It remains to be investigated whether this function occurs through direct interaction of VPS13 with SGS3‐bodies body components or indirectly. Our interaction assays supported the hypothesis of direct interaction of VPS13 with at least SGS3, but other complementary scenarios, such as a possible role of VPS13 in indirectly contributing to SGS3 bodies stability, cannot be completely ruled out. For instance, in Arabidopsis VPS13 might be involved in counteracting the autophagic degradation of siRNA bodies. Autophagy of RDR6/SGS3 siRNA bodies has been demonstrated to be a means of repressing PTGS, a strategy evolved by viruses to bypass host antiviral silencing activity (Field et al., 2021; Tong et al., 2021). The VPS13 protein shares key structural features with the autophagy‐related gene (Atg2), and homologs of VPS13 have been implicated in autophagosome biogenesis from the ER (Dabrowski et al., 2023). The multiple roles of the miR390‐TAS3‐ARF3 pathway during plant development (Fahlgren et al., 2006; Adenot et al., 2006; Marin et al., 2010; Su et al., 2017, 2020), together with the ubiquitarian expression of *VPS13*, suggest it might be a general mechanism required for *ARFs* PTGS (Gala et al., 2021; Koizumi & Gallagher, 2013).

A role of VPS13 inside the nucleus in association with factors involved in gibberellin and cytokinin signaling was recently proposed (Cai et al., 2025). Based on current literature, it is unlikely that VPS13 has a functional role within the nucleus. Studies have consistently shown that VPS13 proteins are primarily associated with membrane contact sites between organelles such as the endoplasmic reticulum, mitochondria, endosomes, and lipid droplets (Dziurdzik & Conibear, 2021). There is no substantial evidence supporting nuclear localization or activity, and the known functions of VPS13 are closely tied to lipid transport and organelle dynamics rather than nuclear processes (Dziurdzik & Conibear, 2021). The model proposed by Cai et al., 2025 was based on the interaction between the VPS13 VAB domain and several transcription factors using Y2H assay, but no interaction or localization was shown *in planta*. In fact, it was already shown that the SHRUBBY domain alone can localize to the nucleus (Koizumi & Gallagher, 2013), but it is unlikely that the full‐length protein might show the same localization—unless the existence of yet unidentified alternative splicing isoforms. Hence, we do believe that VPS13 primarily functions in the cytoplasm by stabilizing complexes in different contexts; in the light of this, we propose VPS13 as a link between SGS3 bodies and ER membranes.

The involvement of VPS13 in the regulation of tasiR‐ARF processing might open new perspectives for the study of reproductive phenotypes associated with *vps13* mutation in other organisms. In fact, it has already been reported that VPS13 has a pivotal role in reproductive processes; these include pro‐spore membrane formation in yeast (Park & Neiman, 2012); oogenesis in *Drosophila* (Faber et al., 2020); male gamete development in mice, and sperm development in humans (Da Costa et al., 2020). Furthermore, our study might help in developing novel approaches in the research related to human neurodegenerative diseases associated with *VPS13* mutations.

## MATERIAL AND METHODS

### Plant material and growth conditions

*Arabidopsis thaliana* plants, Columbia‐0 (Col‐0) ecotype, were used for the experiments. Seeds were sown in soil and then stored at 4°C in the dark for 2 days before moving them to short day (SD) with 8 h of light and 16 h of dark. After a couple of weeks, plants were transferred to long day (LD), with 16 h of light per day. *vps13‐1* (SALK_051137), *pLC2:nlsYFP* (pAt5g40730:nls‐vYFP), *pKNU:nslYFP* (Tucker et al., 2012), *pWOX2:CENH3‐GFP* (De Storme et al., 2016), *pARF3:ARF3‐GFP* (Simonini et al., 2016), and *pASY3:ASY3‐GFP* (Yang et al., 2019) were already described and kindly provided by Matthew Tucker, Nico De Storme, Lars Østergaard, and Arp Schnittger, respectively. The *vps13/+* and *pSPL:VPS13as* were crossed with the marker lines (previously described), and three homozygous F2 plants were analyzed for expression. *sgs3‐14* T‐DNA insertion line (SALK_001394) and *sgs3‐12* EMS line were already described (Peragine et al., 2004). *vps13/+* plants were transformed by floral dip with *pSGS3:SGS3‐GFP* construct (Su et al., 2020) (kindly provided by Xuemei Chen), and three homozygous T2 plants were analyzed for expression.

### Generation of *pSPL:VPS13as:3′SPL* lines

To generate the antisense transcript of *VPS13*, two specific primers were designed to amplify a region of 216 bp of the *VPS13* coding sequence (see Table S2); the obtained fragment was cloned into pDONR207 (Life Technologies). A MultiSite Gateway^®^ approach was then exploited to generate the final vector, combining the entry plasmid (see above) with the pDONR P4‐P1r 5′ UTR SPL and the pDONR P2r‐P3 3′ UTR SPL (Mendes et al., 2020) and subsequently transferring it to the pH7m34GW destination vector (Life Technologies). Wild‐type plants were transformed by floral dip. T1 plants were selected for hygromycin resistance. The presence of the construct was detected by genotyping (see Table S2).

### Morphological analysis of flower structures and siliques

Images of inflorescences, pistils, and siliques were acquired using a Leica^®^ MZ6 stereomicroscope and processed using Axiovision (version 4.1) software. Seed set was analyzed using a stereomicroscope (Leica MZ6); 12–14 days after pollination (DAP), siliques were collected from three different plants from wild‐type and *vps13*. Fruits were placed onto glass slides using double‐sided adhesive tape and their valves were opened using syringe needles.

### Optical microscopy

Cleared ovules were analyzed using DIC microscopy (Zeiss Axiophot D1 × 63) to assess ovule morphologies and alterations. Pictures were acquired with a Zeiss Axiocam MRc5 camera and Axiovision (version 4.1) software. DIC microscopy was used to observe the percentage of MMCs in finger‐like ovules in mutant lines and in the wild‐type. The statistical significance was analyzed using the Student's unpaired *t*‐test.

### Co‐localization in *Nicotiana benthamiana* leaves

VPS13‐C‐terminal domain and SGS3 CDS were first cloned into pDONR207 (Life Technologies) and subsequently transferred to the pB7FWG2 and pB7RWG2 vectors using Gateway recombination for GFP and RFP C‐terminal fusion respectively. The pAM647‐35S:RFP‐AGO7 and pABM168‐35S:RFP‐RDR6 were purchased from the Alexis Maizel collection at the European Plasmid Repository (EPR), while pMDC32‐Kar2‐CFP‐HDEL was purchased from Addgene. Co‐localization assays were performed by injecting *Agrobacterium* expressing viral suppressor p19/experimental constructs. The abaxial surfaces of infiltrated tobacco (*Nicotiana benthamiana*) leaves were imaged 4 days after inoculation at the confocal microscope. Co‐localization has been evaluated using the Fiji ImageJ software, using the JACoP plugin (Bolte & Cordelières, 2006). Overlapping of the signals is presented using the Manders' M1 and M2 coefficients (Manders et al., 1993).

### Confocal microscopy

Confocal laser scanning microscopy of ovules stained with SR2200 was performed on a Nikon Eclipse Ti2 inverted microscope, equipped with a Nikon A1R+ laser scanning device (http://www.nikon.com/). Images were acquired by a CFI Apo Lambda 40XC LWD WI (Numerical Aperture (NA) 1.15). NIS‐Elements (Nikon; http://www.nis‐elements.com/) was used as a platform to control the microscope. Non‐denoised images were analyzed using NIS‐Elements and Fiji. SR2200 was excited with a 405 nm laser line and emission was detected between 415 and 476 nm, whereas eYFP and eGFP were excited at 488 nm and detected at 498–530 nm. Glasses were prepared using a stereomicroscope; for the observation of ovules, pistils were excised from the flowers and covered by a drop of RS2200 solution (0.1% v/v; kept in the dark).

### SGS3‐GFP bodies quantification

For the SGS3‐GFP bodies quantification in wild‐type and *vps13* mutant background in ovules, *vps13/+* heterozygous plants were transformed, via *Agrobacterium*, with the *pSGS3:SGS3‐GFP*. Ovules spanning from stages 2‐I to 2‐III, collected from segregating wild‐type and *vps13* homozygous plants expressing *pSGS3:SGS3‐GFP*, were visualized through confocal microscopy. SGS3‐GFP bodies quantification has been performed with Fiji. Briefly, background noise in the GFP channel was removed with the Subtract Background process. Images were then processed with the difference of Gaussians method (doi:10.1088/1742‐6596/490/1/012020). Gaussian Blur filter with different strengths was applied to the images. Images with different levels of blurring were then subtracted with the Image Calculator tool to obtain a resulting image in which points of discontinuity (SGS3‐GFP foci) are detected. Images were then thresholded with the Otsu method. Individual SGS3‐GFP foci in ovule nucellus were counted with the Analyze Particle tool.

### Total RNA extraction, nuclear RNA extraction, and gene expression analysis

Total RNA from inflorescences was extracted with phenol:chloroform:isoamyl‐alcohol and precipitated using lithium chloride. RNA samples were treated for gDNA contamination and retrotranscribed with iScript™ gDNA Clear cDNA Synthesis Kit (Bio‐Rad Laboratories). Transcripts were detected using a SYBR Green Assay (iQ SYBR Green Supermix; Bio‐Rad Laboratories) using *UBIQUITIN10* as a housekeeping gene. Assays were performed in triplicate using a Bio‐Rad iCycler iQ Optical System (software v.3.0a). The expression of different genes was analyzed using specific oligonucleotide primers (Table S2). Total and nuclear RNA were extracted from shoots 1 week after bolting. Each of the two biological replicates was composed of the aerial parts of at least five different plants. After sampling, shoots were immediately frozen with liquid nitrogen and ground into a powder. To obtain total and nuclear RNA samples for each biological replicate, 50 mg of powder was separated and dissolved into the RNA extraction buffer (100 mm TRIS–HCl pH8, 100 mm LiCl, 10 mm EDTA, and 1% SDS).

The remaining part of the powder was processed for nuclei isolation by solubilization into buffer EB1 (10 mm TRIS–HCl pH8; 10 mm MgCl_2_; 1 mm EDTA; 5 mm β‐mercaptoethanol; Roche cOmpleteTM mini protease inhibitor cocktail; 0.4 m sucrose), and filtration through Millipore Miracloth. After centrifugation, the pellet was resuspended into EB2 (10 mm TRIS–HCl pH8; 10 mm MgCl_2_; 1 mm EDTA; 5 mm β‐mercapto‐ethanol; Roche cOmpleteTM Mini; 0.25 m sucrose; 1% TritonX‐100) to lysate organelles but not nuclei. After centrifugation, the pellet was resuspended in EB3 (10 mm TRIS–HCl pH8; 2 mm MgCl_2_; 1 mm EDTA; 5 mm β‐mercapto‐ethanol; cOmpleteTM Mini; 1.7 m sucrose; 0.15% TritonX‐100). The mixture was then loaded on top of an equal volume of EB3 buffer creating two separate layers. After centrifugation, the nuclei containing pellet was resuspended in nuclei lysis buffer (50 mm TRIS–HCl pH8; 10 mm EDTA; cOmpleteTM Mini; 1% SDS). For both nuclei and total RNA samples, RNA was extracted according to the phenol: chloroform: isoamyl‐alcohol procedure and precipitated with LiCl. An equal amount of RNA for each sample was used for cDNA synthesis. *TAS3* expression between wild‐type and *vps13* in total or nuclear samples was evaluated by real‐time PCR using ACTIN8 as a housekeeping gene.

### Stem‐loop RT‐PCR

Stem‐loop RT‐PCR for detecting the expression of mature miRNA was carried out following the method previously described (Varkonyi‐Gasic et al., 2007). miRNA‐specific stem‐loop RT primers, and forward and reverse primers for individual miRNAs were designed using the last 3′ six nucleotides of a miRNA sequence as the antisense overhang, and the miRNA‐specific forward primer was designed to contain the remaining 5′ sequences (see Table S2). One microgram of total RNA and 1 μl of miRNA‐specific stem‐loop RT primer each (1 μM) for each sample were used for cDNA synthesis using the SuperScript^®^ II First‐Strand Synthesis System for RT‐PCR (Life Technologies, Carlsbad, CA, USA). RT reactions were incubated for 30 min at 16°C, followed by pulsed RT of 60 cycles at 30°C for 30 s, 42°C for 30 s, and 50°C for 1 s. Then, miRNA qRT‐PCR assays were carried out using a Bio‐Rad CFX96 real‐time thermal cycler with the following program: incubation at 94°C for 2 min, followed by 20–40 cycles of 94°C for 15 s and 60°C for 1 min. Endogenous small RNA 5S amenable to the miRNA assay design was used for the normalization (Varkonyi‐Gasic et al., 2007).

### Extraction, cDNA library preparation, and sequencing for total RNA‐seq and short RNA‐seq

Total RNA was extracted using mirVana™ miRNA Isolation Kit, with phenol by Thermofisher. RNA samples were treated for gDNA contamination using the Ambion DNA‐free™ DNA Removal Kit and retrotranscribed with iScript™ gDNA Clear cDNA Synthesis Kit (Bio‐Rad Laboratories). Total RNA was extracted from three biological replicates (0.5 g) from both wild‐type and *vps13* mutant inflorescences. RNA integrity was analyzed by gel electrophoresis and by spectrophotometry before submission to the sequencing facility of the Novogene company, https://www.novogene.com/us‐en/. Novogene had performed RNA sample quality control, RNA and small RNA library preparation, and sequencing by NovaSeq SE50 (150 bp and 50 bp single‐read, 20 m raw reads per sample, respectively).

### Differential expression analysis of small RNAs

Raw sequence data were processed with cutAdapt (Martin, 2011) with default parameters to remove adapters from inserts of between 14 and 38 nt in length. Trimmed reads were mapped to *Arabidopsis thaliana* mature miRNA sequences and phasiRNAs/tasiRNAs, as available from the sRNAanno database (Chen et al., 2021), using bowtie (Langmead et al., 2009) with the following parameters: ‐n 0 ‐L 17–best ‐strata. Only miRNAs and phasiRNAs/tasiRNAs with a read count higher than 10 in at least one sample were considered in the differential expression analysis. Differential expression analyses were performed by the quasi‐likelihood negative binomial generalized log‐linear model test (glmQLFTest) implemented by edgeR (Robinson et al., 2010). Only genes showing an FDR <0.05, and at least a twofold change in expression (|log2(FoldChange)| ≥ 1), and to which 50 or more reads were assigned, were considered differentially expressed.

### *In situ* hybridization assay

Arabidopsis flowers were collected, fixed, and embedded in paraffin, as described by Galbiati et al. (2013). Plant tissue sections were probed with a specific *VPS13* antisense probe (see Table S2). Signal specificity was tested by using a *VPS13* sense probe (Figure S1). Hybridization and immunological detection were executed as described previously by Galbiati et al. (2013).

### Machine‐learning‐based prediction of the fold using AlphaFold2

Protein structure predictions were performed using AlphaFold2 (Evans et al., 2021; Jumper et al., 2021) v2.3.0. Initial sequences were obtained via Uniprot, with the accession codes F4KIH9 (VPS13), Q9LDX1 (SGS3), and Q9SG02 (RDR6). The multiple sequence alignment consisted of 7357, 698, and 6715 sequences for VPS13, SGS3, and RDR6, respectively. The filtered sequence coverage for the complexes is in Figure 7. All the predictions were performed on the multimer‐v3 weights, performing 20 recycles. All the predictions were performed with a machine equipped with a Nvidia A100 GPU. For all the systems, we generated five models, with five different predictions per model, and all the models obtained are available in the supporting information. All the data used by the algorithm (uniref, pdb, and uniprot databases) were downloaded on January 15, 2023.

### Yeast two‐hybrid assay

To test the interaction between VPS13‐VAB domain and SGS3, RDR6, and AGO7, the partial or complete CDS were cloned into pDONR207 (Life Technologies) and transferred to pGBKT7 and pGADT7‐rec (Clontech) by Gateway recombination. The two‐hybrid assay was performed in AH109 yeast strain at 28°C. After transformation, yeast cells were selected on yeast synthetic dropout medium lacking leucine, tryptophan, and histidine and supplemented with different concentrations of 3‐aminotriazole (1, 2.5, 5 mm of 3‐AT, Sigma). Primers used for cloning are listed in Table S2.

### FRET‐FLIM assay

FRET‐FLIM was performed as described in Cavalleri et al. (2026). CDS sequences of VPS13 VAB and C‐terminal domains, as well as SGS3, were cloned in pDONR207 (Life Technologies) and then transferred to the pB7FWG2 and pB7RWG2 vectors using Gateway recombination for eGFP and RFP1 C‐terminal fusion respectively. The constructs obtained were used to infiltrate *Nicotiana benthamiana* leaves. GFP was excited with a 485 nm pulsed laser, and its lifetime was measured by taking advantage of the SymPhoTime 64 software (PicoQuant, https://www.picoquant.com/).

Lifetime measurements were normalized using coumarin6 (0.1 mm EtOH 100%) lifetime acquisition as a reference, by taking advantage of the FLIM‐Phasor analysis software (Vallmitjana et al., 2025; FlimLabs, https://www.flimlabs.com). Lifetime LUT images were generated by the SymPhoTime 64 software. Single channel acquisition is provided in Figure S4. Primers used for cloning are listed in Table S2.

## AUTHOR CONTRIBUTIONS

Conceptualization: LC, PVD, DR, VB, and MC. Data curation: MC and MCh. Formal Analysis: MC, MCh, RC, and CC. Funding acquisition: LC. Investigation: MC, RP, CB, LeC, AC, FR, VB. Methodology: MC and CC. Project administration: LC and RO. Resources: LC and RO. Supervision: LC and MC. Visualization: MC and RP. Writing—original draft: MC and RP. Writing—review and editing: MC, RP, CB, and LC.

## CONFLICT OF INTEREST

The authors declare that they have no conflict of interest.

## Supporting information


**Figure S1.** (A) *VPS13*, *KNUCKLES* (*KNU*), and *SPOROCYTELESS* (*SPL*) expression in MMC, L2, and L1 layers of ovules from the single‐cell dataset of Hou et al. (2021). Expression is shown in UMI counts. (B) Analysis of ovule abortion in nine independent *pSPL:VPS13as* T1 lines; lines 5, 6, and 7 were selected for further analysis. *n* = 10 siliques from each plant. (C) Percentages of wild‐type, *pSPL:VPS13as* and *vps13* ovules showing: multiple MMC‐like cells, MMCs that expressed the *pKNU:nlsYFP* marker, developmental block in FG1 or FG2 stages and final ovule abortion in siliques post‐anthesis. *n* = 18 pistils from six different plants. (D) 3D reconstruction of *Z*‐stacks from *pWOX2:CENH3‐GFP* ovule in wild‐type and *vps13* at functional megaspore stage. Abbreviations: FM, functional megaspore; s, spore; ds, degenerating spore. (E) Ovule sections hybridized with a VPS13‐sense probe.
**Figure S2.** (A–D) Co‐localization assay of the endoplasmic reticulum markers HDEL‐CFP with VPS13‐C‐RFP (A), SGS3‐RFP (B), RDR6‐RFP (C) and AGO7‐RFP (D). Graphs report the percentage of co‐localization based on the calculation of Manders coefficients. (E) As negative controls in all *Nicotiana benthamiana* localization experiments we performed infiltration with infiltration media without *Agrobacterium*, and we checked levels of CFP, RFP, and GFP auto fluorescence.
**Figure S3.** (A–C) Yeast two‐hybrid assay to check interaction of VPS13 VAB domain with SGS3 (A), AGO7 (B), and RDR6 (C). The strength of interaction was tested with different concentrations of 3AT on ‐L ‐W ‐H selective media and four serial dilutions of yeast cells.
**Figure S4.** (A–D) LUT images of VPS13‐C‐GFP alone, VPS13‐C‐GFP with SGS3‐RFP, SGS3‐GFP, and SGS3‐GFP with VPS13‐C‐RFP, respectively. (E–T) Single‐channel pictures for the different combinations of interaction tested. (E–F) SGS3‐GFP alone, (G–H) SGS3‐GFP with VPS13‐VAB‐RFP; (I–J) VPS13‐VAB‐GFP alone; (K–L) VPS13‐VAB‐GFP with SGS3‐RFP; (M–N) VPS13‐C‐GFP alone; (O–P) VPS13‐C‐GFP with SGS3‐RFP; (Q–R) SGS3‐GFP alone; (S–T) SGS3‐GFP with VPS13‐C‐RFP. (U) Graph showing GFP lifetime measured in cells expressing VPS13‐C‐GFP or SGS3‐GFP alone compared with those measured in cells co‐expressing VPS13‐C‐GFP or SGS3‐GFP together with SGS3‐RFP or VPS13‐C‐RFP, respectively. (V) Graph showing percentage of multiple MMC‐like cells in *sgs3‐14* and *sgs3‐12* compared with the wild‐type. (*) *P*‐value, <0.05; (**) *P*‐value, <0.01. Scale bars, 10 μm.

**Table S1.** List of differentially expressed genes (DEGs), differentially expressed miRNAs (DE miRNAs) and differentially expressed phasiRNAs and tasiRNAs (DE tasiRNAs and phasiRNAs) in *vps13* versus wild‐type RNA‐seq and sRNA‐seq on inflorescences.

**Table S2.** List of oligonucleotides used in this study.

## Data Availability

RNA‐seq raw data have been deposited to the NCBI Sequence Read Archive (SRA) under the accession PRJNA1444070. Small RNA‐seq data have been deposited to the NCBI Sequence Read Archive (SRA) under the accession PRJNA1443689.
