# Supplementary material for: VPS13 has an important role in female germline development in Arabidopsis
Source: Plant J. 2026 May 10;126:e70898. doi: 10.1111/tpj.70898 (PMC13157975; doi:10.1111/tpj.70898)
Supplement: Supplementary file 1 — Figure S1. (A) VPS13, KNUCKLES (KNU), and SPOROCYTELESS (SPL) expression in MMC, L2, and L1 layers of ovules from the single‐cell dataset of Hou et al. (2021). Expression is shown in UMI counts. (B) Analysis of ovule abortion in nine independent pSPL:VPS13as T1 lines; lines 5, 6, and 7 were selected for further analysis. n = 10 siliques from each plant. (C) Percentages of wild‐type, pSPL:VPS13as and vps13 ovules showing: multiple MMC‐like cells, MMCs that expressed the pKNU:nlsYFP marker, developmental block in FG1 or FG2 stages and final ovule abortion in siliques post‐anthesis. n = 18 pistils from six different plants. (D) 3D reconstruction of Z‐stacks from pWOX2:CENH3‐GFP ovule in wild‐type and vps13 at functional megaspore stage. Abbreviations: FM, functional megaspore; s, spore; ds, degenerating spore. (E) Ovule sections hybridized with a VPS13‐sense probe. Figure S2. (A–D) Co‐localization assay of the endoplasmic reticulum markers HDEL‐CFP with VPS13‐C‐RFP (A), SGS3‐RFP (B), RDR6‐RFP (C) and AGO7‐RFP (D). Graphs report the percentage of co‐localization based on the calculation of Manders coefficients. (E) As negative controls in all Nicotiana benthamiana localization experiments we performed infiltration with infiltration media without Agrobacterium, and we checked levels of CFP, RFP, and GFP auto fluorescence. Figure S3. (A–C) Yeast two‐hybrid assay to check interaction of VPS13 VAB domain with SGS3 (A), AGO7 (B), and RDR6 (C). The strength of interaction was tested with different concentrations of 3AT on ‐L ‐W ‐H selective media and four serial dilutions of yeast cells. Figure S4. (A–D) LUT images of VPS13‐C‐GFP alone, VPS13‐C‐GFP with SGS3‐RFP, SGS3‐GFP, and SGS3‐GFP with VPS13‐C‐RFP, respectively. (E–T) Single‐channel pictures for the different combinations of interaction tested. (E–F) SGS3‐GFP alone, (G–H) SGS3‐GFP with VPS13‐VAB‐RFP; (I–J) VPS13‐VAB‐GFP alone; (K–L) VPS13‐VAB‐GFP with SGS3‐RFP; (M–N) VPS13‐C‐GFP alone; (O–P) VPS13‐C‐GFP with SGS3‐RFP; (Q [file TPJ-126-0-s002.docx]

**SUPPLEMENTAL FIGURES**

**
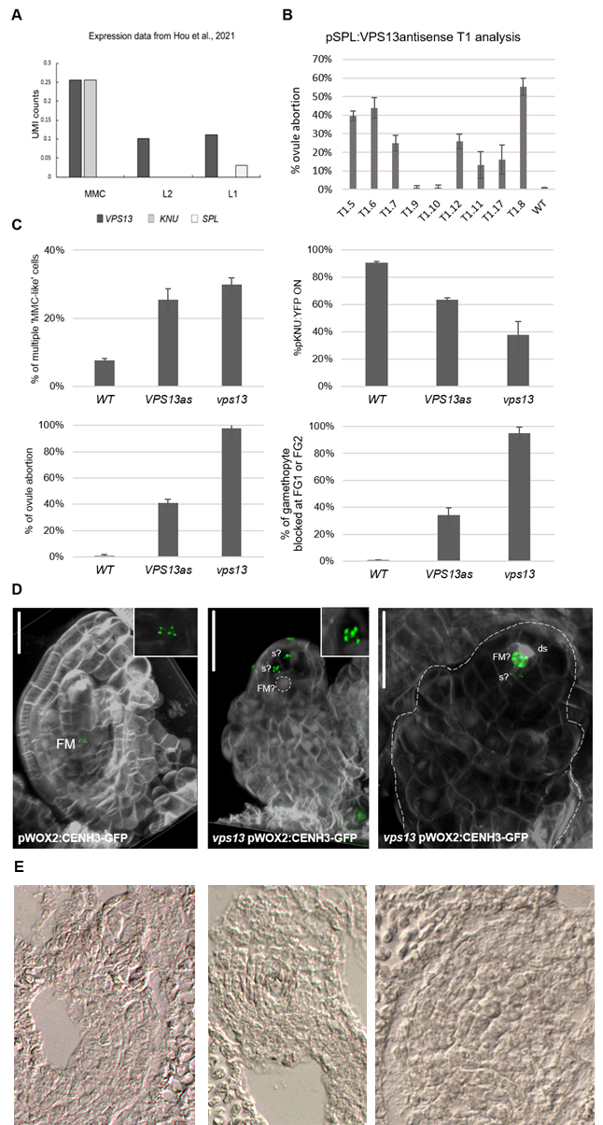
**

**Supplemental Figure 1.** (A) *VPS13*, *KNUCKLES* (*KNU*), and *SPOROCYTELESS* (*SPL*) expression in MMC, L2, and L1 layers of ovules from the single cell dataset of Hou et al., (2021). Expression is shown in UMI counts. (B) Analysis of ovule abortion in nine independent *pSPL:VPS13as* T1 lines; lines 5, 6 and 7 were selected for further analysis. n=10 siliques from each plant. (C) Percentages of wild-type, *pSPL:VPS13as* and *vps13* ovules showing: multiple MMC-like cells, MMCs that expressed the *pKNU:nlsYFP* marker, developmental block in FG1 or FG2 stages and final ovule abortion in siliques post-anthesis. n=18 pistils from six different plants. (D) 3D reconstruction of *Z*-stacks from *pWOX2:CENH3-GFP* ovule in wild-type and *vps13* at functional megaspore stage. Abbreviations: FM, functional megaspore; s, spore; ds, degenerating spore. (E) Ovule sections hybridized with a VPS13-sense probe.


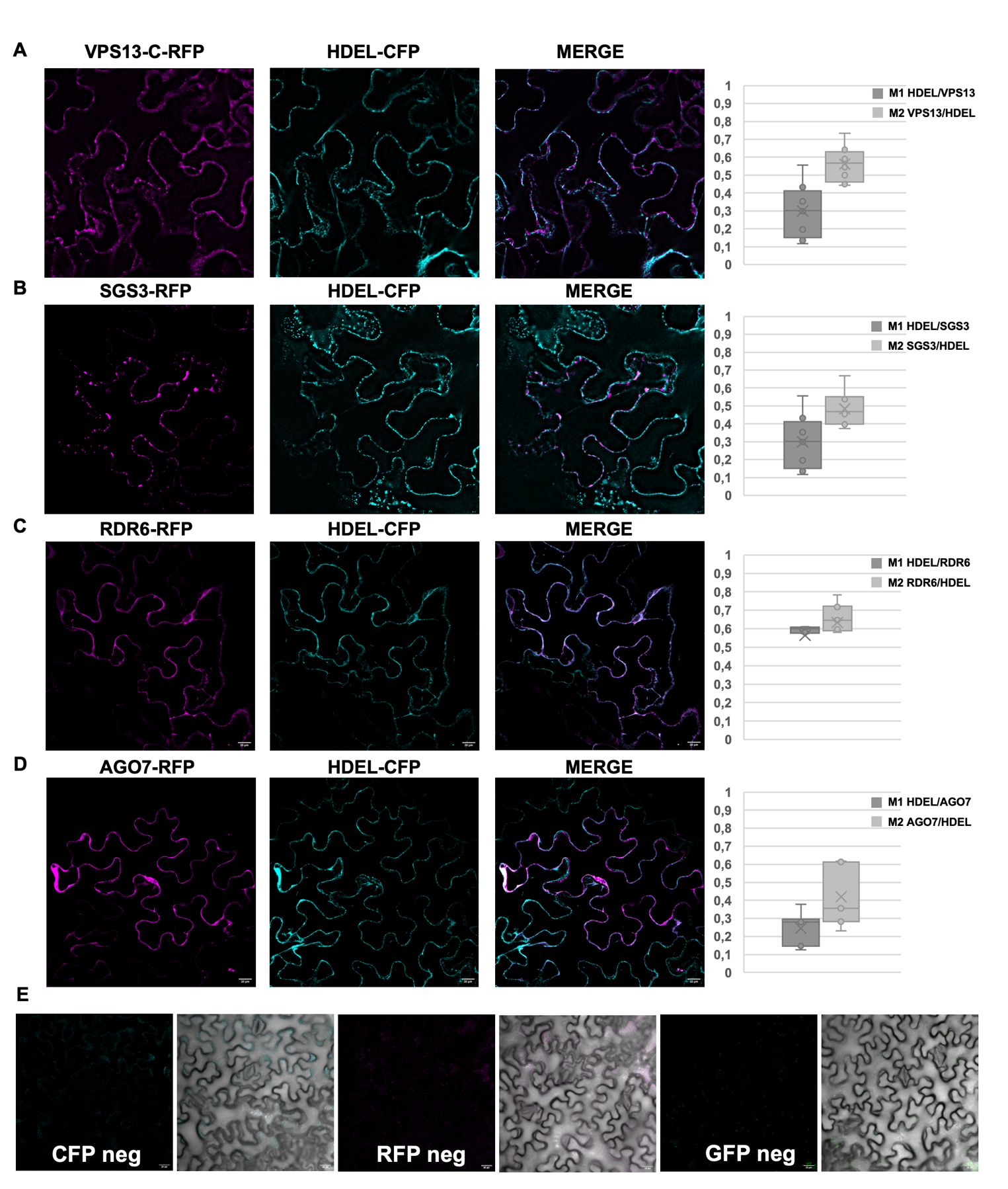


**Supplemental Figure 2.** (A-D) Colocalization assay of the endoplasmic reticulum markers HDEL-CFP with VPS13-C-RFP (A), SGS3-RFP (B), RDR6-RFP (C) and AGO7-RFP (D). Graphs report the percentage of co-localization based on the calculation of Manders coefficients. (E) As negative controls in all *Nicotiana benthamiana* localization experiments we performed infiltration with infiltration media without agrobacterium, and we checked levels of CFP, RFP, and GFP auto fluorescence.


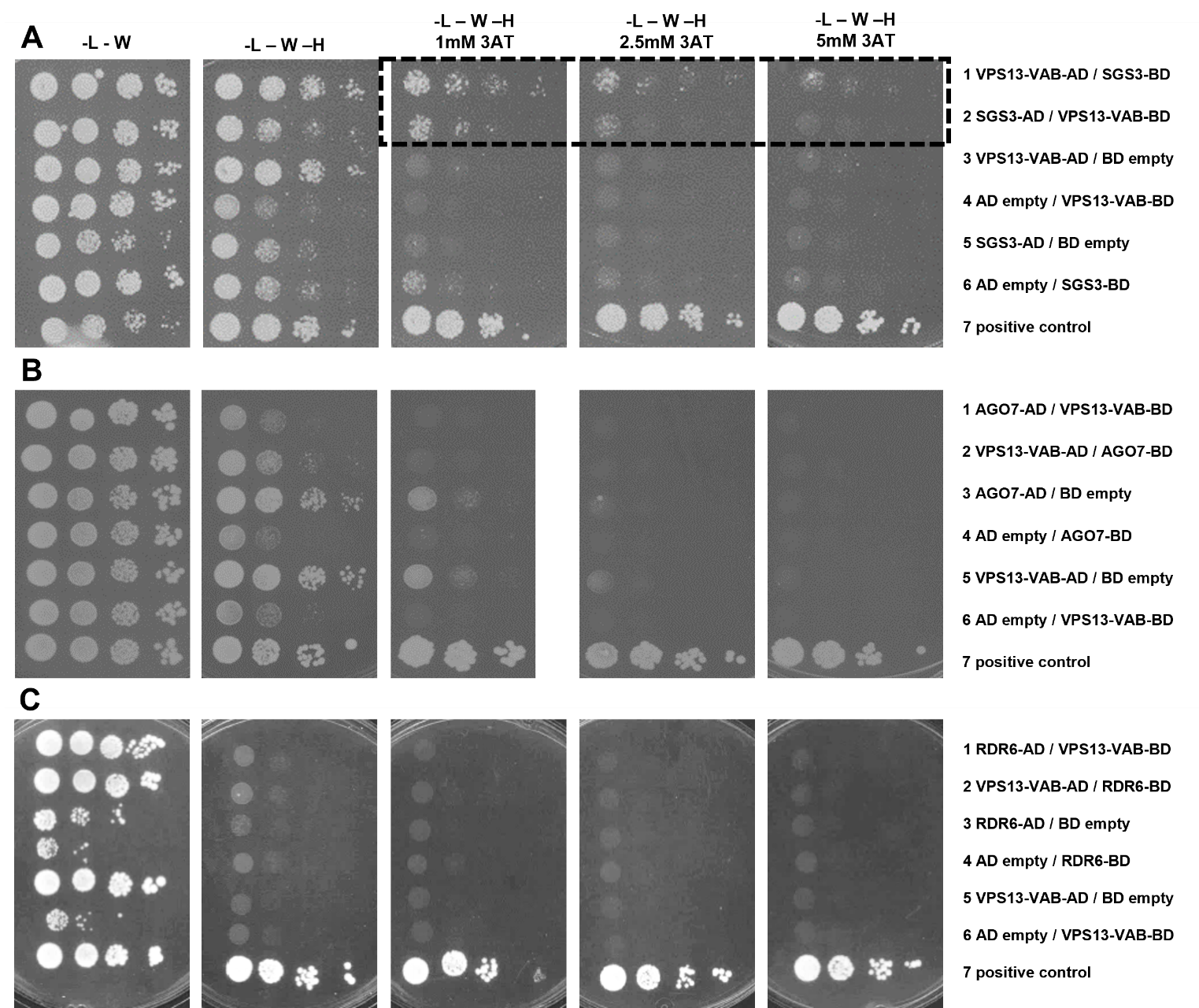


**Supplemental Figure 3.** (A-C) Yeast two hybrid assay to check interaction of VPS13 VAB domain with SGS3 (A), AGO7 (B) and RDR6 (C). The strength of interaction was tested with different concentrations of 3AT on -L -W -H selective media and four serial dilutions of yeast cells.

**
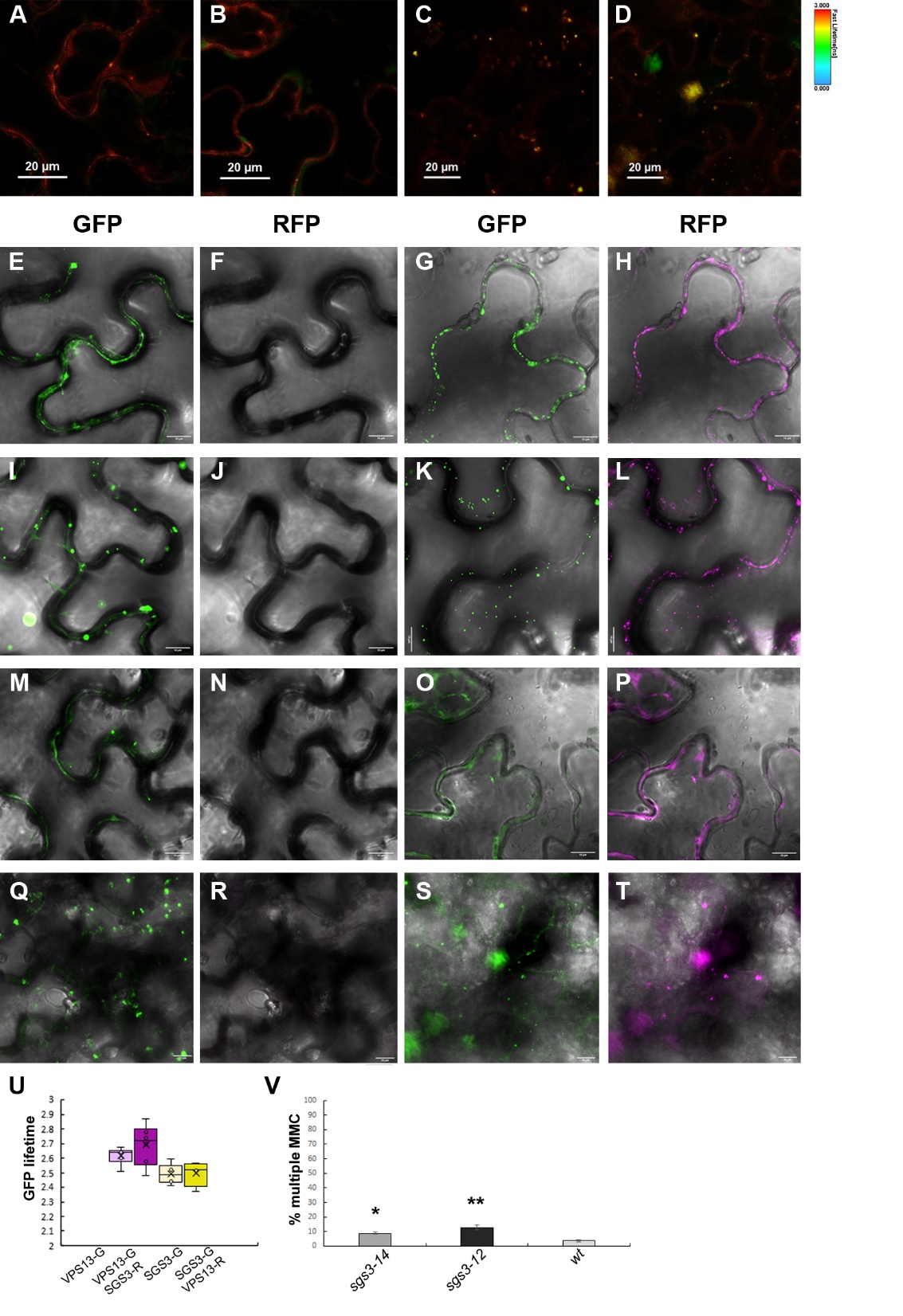
**

**Supplemental Figure 4.** (A-D) LUT images of VPS13-C-GFP alone, VPS13-C-GFP with SGS3-RFP, SGS3-GFP and SGS3-GFP with VPS13-C-RFP, respectively. (E-T) Single channel pictures for the different combinations of interaction tested. (E-F) SGS3-GFP alone, (G-H) SGS3-GFP with VPS13-VAB-RFP; (I-J) VPS13-VAB-GFP alone; (K-L) VPS13-VAB-GFP with SGS3-RFP; (M-N) VPS13-C-GFP alone; (O-P) VPS13-C-GFP with SGS3-RFP; (Q-R) SGS3-GFP alone; (S-T) SGS3-GFP with VPS13-C-RFP. (U) Graph showing GFP lifetime measured in cells expressing VPS13-C-GFP or SGS3-GFP alone compared with those measured in cells co-expressing VPS13-C-GFP or SGS3-GFP together with SGS3-RFP or

VPS13-C-RFP, respectively. (V) Graph showing percentage of multiple MMC-like cells in *sgs3-14* and *sgs3-12* compared to the wild-type. (*) p-value, < 0.05; (**) p-value, <0.01. Scale bars, 10 µm.
