# Supplementary material for: VPS13 has an important role in female germline development in Arabidopsis
Source: Plant J. 2026 May 10;126:e70898. doi: 10.1111/tpj.70898 (PMC13157975; doi:10.1111/tpj.70898)
Supplement: Supplementary file 3 — Table S2. List of oligonucleotides used in this study. [file TPJ-126-0-s001.docx]

| **Primer sequence** | **Description** |
| --- | --- |
| CATTGGTGTTGAAGACATGC | *vps13*-1 fw genotyping on wt locus |
| ATTTTGCCGATTTCGGAAC | *vps13*-1 rv genotyping on wt locus |
| ATTTTGCCGATTTCGGAAC | LBb3 |
| ATGGTGTATGGAACTGGAGA | VPS13 ISH probe fw |
| CTTTTCCCAGTTTC | VPS13 ISH probe rv |
| TAATACGACTCACTATAGGGCTTTTCCCAGTTTC | VPS13 ISH rv + T7 |
| TAATACGACTCACTATAGGG ATGGTGTATGGAACTGGAGA | VPS13 ISH probe + T7 |
| GGGGACAAGTTTGTACAAAAAAGCAGGCTcgACACACTCAACAAGTCCTCTG | VPS13as cloning fw + AttB1 |
| GGGGACCACTTTGTACAAGAAAGCTGGGTgAGAGAAATCGGTTTGGAGAGTG | VPS13as cloning rv + AttB2 |
| gctttcgtctttacctcttccc | pSPL:VPS13as genotyping fw on PSPL |
| AGAGAAATCGGTTTGGAGAGTG | pSPL:VPS13as genotyping fw on VPS13 |
| GTCGTATCCAGTGCAGGGTCCGAGGTATTCGCACTGGATACGACGGCGCT | Mir390a/b for stem-loop PCR |
| GCGGCGGAAGCTCAGGAGGGAT | Mir390a/b fw for stem-loop |
| GTGCAGGGTCCGAGGT | Universal primer for stem-loop |
| GTCGTATCCAGTGCAGGGTCCGAGGTATTCGCACTGGATACGACATCCTG | 5S RNA stem-stem loop RNA |
| CGGAAAAATAGCTCGACGC | 5S RNA stem-stem loop RNA |
| tagagaagaatctgtaaagctcagga | premiR390a fw for qRT-PCR |
| agaagagccaatgaaactcagg | premiR390a rv for qRT-PCR |
| tggctcaccagtgctgtatg | premiR390b fw for qRT-PCR |
| gatggatagcgccaacagat | premiR390b rv for qRT-PCR |
| TAAGGAAAACATAACCTCCG | *TAS3* fw for qRT-PCR |
| agaaagagatggggtcttaca | *TAS3* rv for qRT-PCR |
| CAACACTTGTTCGGATGGTG | *ARF3* fw for qRT-PCR |
| CCCACACCAAATGTTCCTCT | *ARF3* rv for qRT-PCR |
| CTGTTCACGGAACCCAATTC | *UBI10* fw for qRT-PCR |
| GGAAAAAGGTCTGACCGACA | *UBI10* rv for qRT-PCR |
| CTCAGGTATTGCAGACCGTATGAG | *ACT8* fw for qRT-PCR |
| CTGGACCTGCTTCATCATACTCTG | *ACT8* rv for qRT-PCR |
| GGGGACAAGTTTGTACAAAAAAGCAGGCTtcATGAGTTCTAGGGCTGGTCC | *SGS3* cloning fw + AttB1 |
| GGGGACCACTTTGTACAAGAAAGCTGGGTgATCATCTTCATTGTGAAGGCC | *SGS3* cloning w/o STOP rv + AttB2 |
| GGGGACCACTTTGTACAAGAAAGCTGGGTgtcaatcatcttcattgtgaaggcc | *SGS3* cloning w/ STOP rv + AttB2 |
| GGGGACAAGTTTGTACAAAAAAGCAGGCTtcATGGTTCAAAGTCGAAAAGTTG | VPS13 VAB domain cloning fw + AttB1 |
| GGGGACCACTTTGTACAAGAAAGCTGGGTcGATAGATAGCAACAATGTCC | VPS13 VAB domain cloning w/o STOP rv + AttB2 |
| GGGGACCACTTTGTACAAGAAAGCTGGGTcCTAGATAGATAGCAACAATGTCC | VPS13 VAB domain cloning w/ STOP rv + AttB2 |
| GGGGACAAGTTTGTACAAAAAAGCAGGCTtcATGATTAGGATTGGTGAATTTGGAC | VPS13 C-terminal domain domain cloning fw + AttB1 |
| GGGGACCACTTTGTACAAGAAAGCTGGGTcAGTGATGTTACTTCGACTTAAAAC | VPS13 C-terminal domain domain w/o STOP rv + AttB2 |
| CTTGAAGTCGATGCCCTTCAGC | pSGS3:SGS3-GFP genotyping fw |
| CAGTGGAAGGGTTTGGGTGA | pSGS3:SGS3-GFP genotyping rv |
| AAATTTGGAGTCCAGAATCGG | *sgs3-14* fw wt band |
| CAAAGCATCGGAATCATTCTC | *sgs3-14* wt and mutant band rv |
| ATTTTGCCGATTTCGGAAC | LBb1.3 SALK-T-DNA |
| CAAATAGCCAGTGGCACTGC | *sgs3-12* fw |
| CAAATAGCCAGTGGCACTGC | *sgs3-12* rv |
